# Nanoparticle‐Based Antiviral Vaccines for Chickens: Recent Advances and Future Perspectives

**DOI:** 10.1155/tbed/2445968

**Published:** 2025-12-11

**Authors:** Peiyang Ding, Litong Xia, Shuoqi Dong

**Affiliations:** ^1^ School of Life Sciences, Zhengzhou University, Zhengzhou, 450001, China, zzu.edu.cn

**Keywords:** chicken vaccines, immune mechanisms, nanotechnology, viral diseases

## Abstract

Viral diseases in chickens, such as avian influenza, Newcastle disease, and infectious bronchitis, cause significant economic losses to the global chicken industry, while the cross‐species transmission risks of avian influenza viruses (AIVs) pose potential threats to public health security. Conventional inactivated vaccines and live attenuated vaccines exhibit limitations in terms of protective coverage, immunization duration, and safety profiles, necessitating the development of novel vaccine strategies. In recent years, the application of nanotechnology has been bringing new opportunities for chicken vaccine development. As innovative vaccine carriers, nanoparticles demonstrate unique advantages through their size effects, surface modifiability, and antigen‐loading capacity, enabling precise regulation of antigen delivery efficiency and enhance innate immune responses via activation of pattern recognition receptors. This review summarizes recent advancements in nanoparticle‐based vaccines for chickens, with particular emphasis on nanocarrier design principles, immunological mechanisms, and protective efficacy. The potential of these systems to improve immune responses and extend protective duration is systematically analyzed, with future research priorities outlined to guide the development of next‐generation chicken vaccines.

**Summary**

• Integrates latest developments in nanoparticle‐based chicken vaccines, bridging nanotechnology and immunology.
• Comprehensive review of innovative principles for various vaccine designs and immune activation mechanisms.
• Proposes solutions, like broad‐spectrum antigens and mucosal delivery, while addressing efficacy, safety, and scalability.
• Integrates multidisciplinary perspectives on efficacy, safety, and practical scalability for global chicken health.

## 1. Introduction

**Figure 1:**
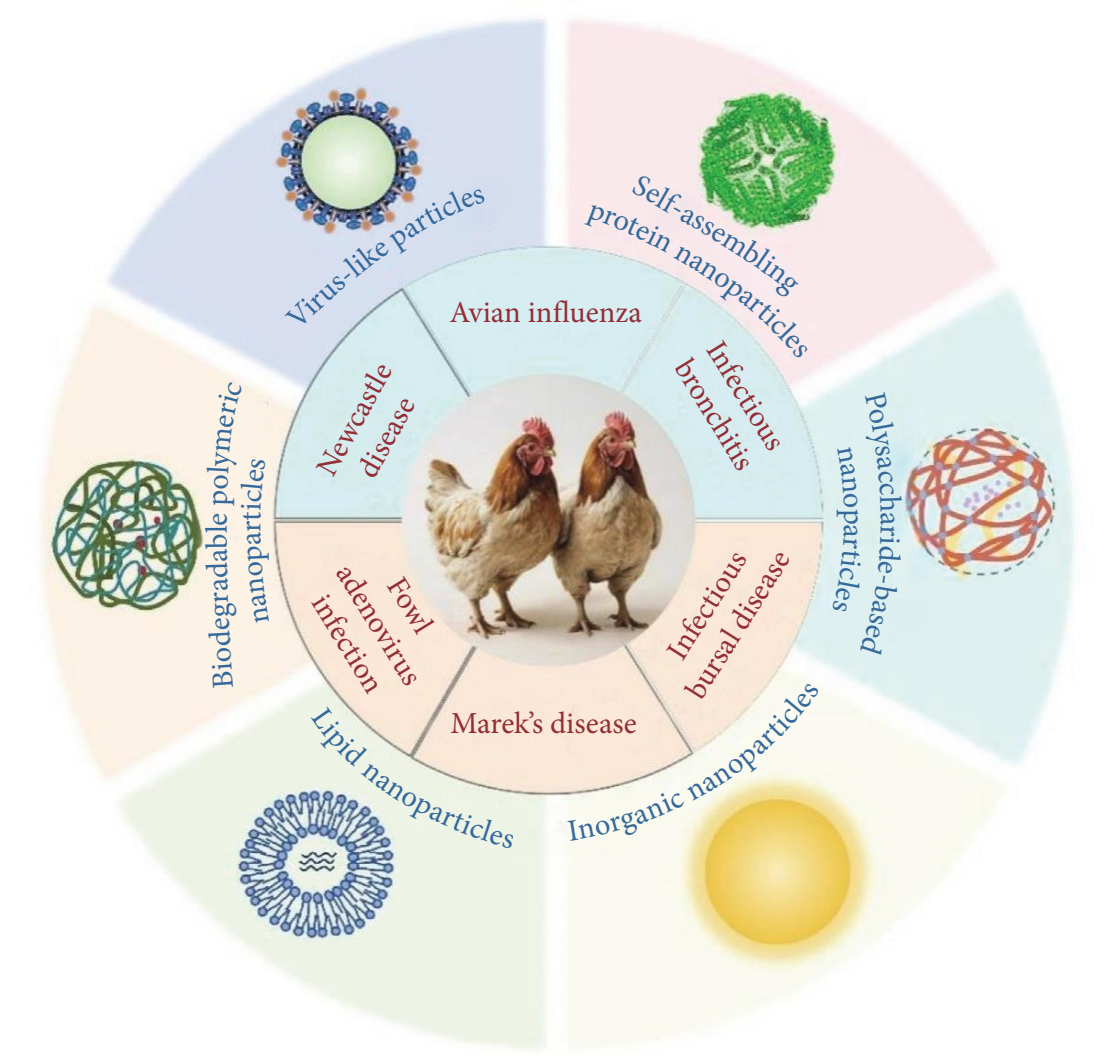
Important chicken viral diseases and types of nanovaccines.

Viral diseases present significant economic and health challenges to the global chicken industry. Traditional vaccines often fall short in terms of cross‐protection, duration of immunity, and safety, which creates a pressing need for innovative solutions [1, 2]. In this context, nanoparticle‐based vaccines have emerged as a promising alternative, utilizing their unique characteristics such as targeted antigen delivery, enhanced immunogenicity, and improved stability to overcome these limitations. This review explores the latest advancements in nanoparticle‐based vaccines for chickens, emphasizing their design strategies, immune mechanisms, and protective efficacy (Figure 1). The discussion underscores the transformative potential of these vaccines in chicken vaccination practices and identifies key research priorities for their further development.

## 2. Major Viral Diseases and Conventional Vaccine

Numerous viral diseases significantly impact the chicken industry, leading to considerable economic losses, prompting the development of vaccines against these pathogens. However, conventional vaccines, whether inactivated or live‐attenuated, often face limitations such as a short duration of immunity, risks of virulence reversion, interference from maternal antibodies, and restricted routes of administration [3]. To address these challenges, future research should focus on identifying broad‐spectrum antigens, designing multivalent vaccines, and optimizing genetic engineering techniques. These efforts aim to overcome serotype and genotype restrictions, achieve long‐lasting cross‐protection, reduce dependence on cold‐chain logistics, and explore innovative mucosal immunization methods [4]. Additionally, Table 1 provides a summary of economically significant viral diseases affecting chickens along with their respective vaccines.

**Table 1:** Major viral diseases in chickens: epidemiology, conventional vaccines, and unmet needs.

Disease	Virus	Epidemiology and clinical signs	Vaccines available	Improvements needed	References
Avian influenza (AI)	Avian influenza virus (AIV) Single strand (−) RNA	Global, sporadic outbreaks High mortality, respiratory distress, cyanosis, edema, drop in egg production	Inactivated whole virus	Cross‐protection against divergent subtypes, thermostable formulations for tropical regions	[5, 6]
Newcastle disease (ND)	Newcastle disease virus (NDV) Single strand (−) RNA	Global, endemic in Asia, Africa, and South America Respiratory distress, greenish diarrhea, nervous signs (tremors and paralysis), high mortality	Live attenuated, inactivated vaccines	Improve heat stability for field use, DIVA‐compatible strategies to distinguish vaccinated from infected birds	[7]
Infectious bronchitis (IB)	Infectious bronchitis virus (IBV) Single strand (+) RNA	Global Respiratory distress (gasping, coughing), reduced egg production, misshapen eggs, nephritis	Live attenuated (mass, 793B), Inactivated multivalent vaccines	Broader coverage of emerging variants, Mucosal immunity enhancement	[8]
Fowl adenovirus infection	Fowl adenovirus (FAdV) Double strand DNA	Global Inclusion body hepatitis (IBH), hydropericardium syndrome (HPS), lethargy, sudden death	Inactivated serotype‐specific vaccines, Live attenuated (limited use)	Cross‐serotype protection, Reduction of vertical transmission risks	[9, 10]
Marek’s disease (MD)	Marek’s disease virus (MDV) Double strand DNA	Global Paralysis, tumors in visceral organs, blindness (ocular form), immunosuppression	Herpesvirus of turkeys (HVT), Bivalent (HVT + SB‐1) vaccines	Protection against “very virulent plus” (vv + MDV) strains, Mitigation of vaccine‐induced viral evolution	[11]
Infectious bursal disease (IBD)	Infectious bursal disease virus (IBDV) Double strand RNA	Global Immunosuppression, edema/bruising of the bursa, diarrhea, high mortality in young chicks	Live attenuated (intermediate plus), Subunit vaccine (VP2)	Reduction of vaccine‐induced immunosuppression, Heat‐stable formulations for global distribution	[12]

### 2.1. RNA Viral Diseases

Several RNA virus‐induced diseases significantly affect the chicken industry, with the Avian influenza virus (AIV), Newcastle disease virus (NDV), and infectious bronchitis virus (IBV) being the most notable. The high mutation rates of RNA viruses create considerable challenges for disease control.

Highly pathogenic AIV, especially subtypes H5N1 and H7N9, attract global attention due to their severe pathogenicity, widespread transmission, and zoonotic risks. Although the H9N2 subtype is less virulent, it still results in substantial economic losses worldwide, particularly as vaccine efficacy declines over time [2, 5]. The frequent antigenic drift in AIV leads to mismatches between vaccines and circulating strains, resulting in a rapid decrease in protection rates and potentially increasing the number of susceptible populations, which exacerbates outbreaks. Current AIV vaccines mainly consist of inactivated formulations that provide strong protection against homologous strains but show limited cross‐reactivity against heterologous strains [6]. Furthermore, the absence of differentiating infected from vaccinated animals (DIVA)‐compatible vaccines complicates surveillance efforts. Future vaccine development should prioritize broad‐spectrum options that target conserved antigens or multivalent formulations, alongside the exploration of mucosal immunization strategies to enhance protective coverage [13–15].

NDV significantly impacts chicken populations, leading to high mortality rates and decreased productivity, with the effectiveness of vaccines differing among various viral strains [16–18]. Traditional live vaccines induce mucosal immunity but carry shedding risks, while inactivated vaccines offer safety advantages but weaker immune responses. The emergence of multiple NDV genotypes has resulted in inconsistent vaccine performance, highlighting the need for research to focus on developing genotype‐matched multivalent vaccines, optimizing vector‐based platforms, and implementing strategies to enhance cellular immunity [7, 19, 20].

IBV is known for its multiple serotypes and primarily affects chickens, leading to significant respiratory and reproductive issues, decreased egg production, and a decline in egg quality, which can often result in high mortality rates [21]. Currently, vaccines available for IBV are based on either live‐attenuated or inactivated formulations that are specifically designed to match the serotypes prevalent in different regions. However, the considerable diversity among serotypes and the limited cross‐protection offered by these vaccines can lead to a significant drop in their effectiveness when there is a mismatch between the vaccine strains and the circulating strains of the virus [22]. To address these challenges, future research and development should concentrate on creating multivalent vaccines, exploring chimeric recombinant designs, or developing universal vaccines that target conserved antigens, which could help to overcome the limitations posed by the various serotypes of IBV [8, 23, 24].

### 2.2. DNA Viral Diseases

DNA viruses continue to pose significant health challenges to global chicken production, primarily through three key pathogens: Fowl adenovirus (FAdV), Marek’s disease virus (MDV), and infectious bursal disease virus (IBDV).

FAdV, which is a double‐stranded DNA virus, mainly affects chicken aged 3–5 weeks, leading to conditions such as pericardial effusion syndrome and hemorrhagic hepatitis [9, 25, 26]. The virus spreads through feces, contaminated water, and respiratory routes, resulting in considerable economic losses for the chicken industry [27, 28]. While current inactivated vaccines are designed to target specific serotypes, particularly FAdV‐4, the emergence of highly pathogenic strains like FAdV‐8 b and the lack of sufficient cross‐serotype protection present ongoing challenges. Therefore, future research should prioritize the development of multivalent inactivated vaccines, subunit vaccines utilizing virus‐like particles (VLPs), or epitope‐based designs to enhance serotype coverage and improve overall vaccine efficacy [10].

MDV, a herpesvirus that spreads through aerosols, leads to the development of lymphomatous tumors after a latent infection. In areas where vaccine coverage is inadequate, the vertical transmission rates of this virus can result in mortality rates exceeding 40% [29–32]. While heterologous vaccines, such as the herpesvirus of Turkey (HVT), are effective in suppressing tumor formation, they do not prevent the infection or shedding of the wild‐type virus. It is important to note that very virulent strains of MDV can sometimes overcome the immune defenses provided by these vaccines. Therefore, current research priorities focus on developing multivalent recombinant vaccines, such as HVT–IBD, creating new adjuvanted formulations, and implementing strategies aimed at boosting cellular immunity to reduce viral shedding [11, 29, 33].

IBDV, a nonenveloped double‐stranded DNA virus that causes significant immunosuppression by targeting and destroying the bursa of fabricius, particularly affecting broilers aged 3–6 weeks, who show high susceptibility and can experience mortality rates of upto 30% [34]. Live‐attenuated vaccines with moderate virulence carry a risk of bursal damage, while inactivated vaccines exhibit enhanced safety but short‐lived immunity [35]. Additionally, the emergence of variant strains has raised concerns as they can evade the protection offered by conventional vaccines. To address these challenges, future research should focus on developing updated vaccine strains that are in line with circulating variants, exploring VLPs‐based or genetically engineered attenuated vaccines, and finding ways to mitigate the risks associated with immunosuppression [12, 36].

## 3. Nanoparticle Vaccine Platforms: Mechanisms and Advantages

**Figure 2:**
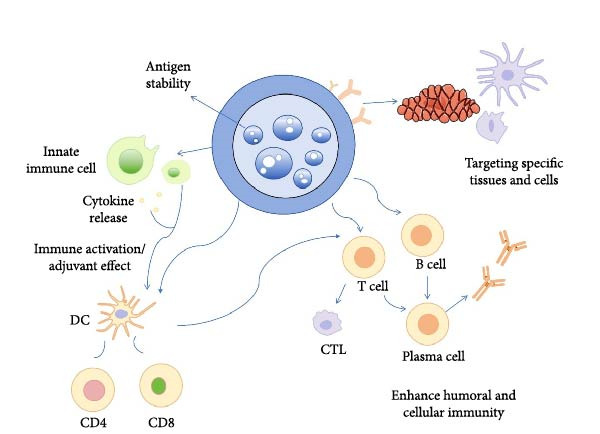
Advantages of nanoparticle vaccines.

Nanoparticle vaccine demonstrate significant advantages in antigen delivery and immune activation due to their unique design strategies [37]. The use of nanoencapsulation technology safeguards antigens from being broken down by enzymes and shields them from environmental factors, which greatly improves their stability. Furthermore, modifying the surface of these nanoparticles allows for targeted delivery, minimizing systemic side effects. In addition to these benefits, nanoparticles possess inherent adjuvant properties that stimulate innate immunity and enhance specific immune responses. By working together to activate both cellular and humoral immunity, these platforms ultimately create a comprehensive immune protection network [38, 39] (Figure 2).

Nanoparticle vaccines significantly improve the in vivo stability of antigens. This stability is achieved by encapsulating antigens within nanoparticles, shielding them from enzymatic degradation and adverse environmental factors. Nanoparticles prolong the antigen half‐life and enhance bioavailability, thereby increasing the intensity and duration of immune responses [40, 41]. Additionally, the physical characteristics of nanoparticles, including their size and surface properties, can be tailored to further enhance both the stability of the antigens and their ability to provoke an immune response [42].

The nanoparticle vaccine allows for targeted delivery to specific cells or tissues through surface modifications. This approach not only enhances the effectiveness of the vaccine but also reduces systemic side effects. By functionalizing nanoparticles with ligands or antibodies, their capacity to identify and attach to particular immune cells is improved, thereby boosting immune responses [43–45].

Nanoparticles play a significant part in activating innate immunity and enhancing adaptive immune responses. They stimulate immune cells to release cytokines, which are vital for cell signaling in the immune system, and improve the presentation of antigens, thereby facilitating T‐cell activation. Some nanoparticles have the ability to directly stimulate natural killer (NK) cells and dendritic cells (DCs), both of which are essential for effective antigen presentation and the priming of T‐cells. Additionally, the adjuvant effect of nanoparticles can be fine‐tuned by adjusting their physical characteristics, including size and shape, to optimize their interaction with the immune system [46–48].

Nanoparticle vaccines concurrently induce cellular and humoral immunity, providing comprehensive immune protection. They enhance cellular immunity by activating CD4^+^ T cells and CD8^+^ T cells, which play crucial roles in identifying and eliminating infected cells. Simultaneously, humoral immunity is bolstered through the activation of B cells, leading to increased antibody production. This dual activation mechanism demonstrates robust efficacy against diverse viral infections, particularly in scenarios requiring rapid and potent immune responses [49–52].

## 4. Application of Nanoparticle Platforms Against Chicken Viruses

The landscape of nanoparticle vaccine platforms is diverse, with each type offering a unique set of advantages tailored to specific immunological and practical needs. VLPs and self‐assembling protein nanoparticles (SAPNs) excel in structurally mimicking pathogens, thereby priming strong, neutralizing antibody‐focused responses. In contrast, lipid nanoparticles (LNPs) and biodegradable polymeric nanoparticles act as versatile “cargo ships,“ ideal for the codelivery of antigens and immunostimulants and for inducing robust T‐cell immunity through sustained release. Polysaccharide‐based systems, particularly chitosan and its derivatives, are front‐runners for inducing mucosal immunity. Meanwhile, inorganic nanoparticles offer potent, tunable adjuvant effects. The subsequent analysis, guided by the framework in Table 2, details how these comparative strengths inform strategic choices against specific chicken viruses.

**Table 2:** Protective efficacy of nanoparticle‐based vaccines against major chicken viral diseases.

Target virus	Antigens	Nanoparticles platform	Adjuvant	Animal and route	Immunogenicity findings	Protection evidence (challenge model)	References
Avian influenza virus (AIV)	HA, NA, M1 (H9N2)	VLPs	None	Chicken, IM, and SQ	Induced high‐titer neutralizing antibodies and virus‐specific IgG	Not assessed	[53]
	HA, NA, M1 (H7N9)	VLPs	Montanide ISA 71 VG	Chicken, IM	Elicited a balanced Th1/Th2 response (increased IL‐2, IL‐4, IFN‐γ)	Complete protection against lethal H7N9 challenge; significantly reduced viral shedding and lung pathology	[54]
	HA, NA, M1 (H5 and H7 bivalent)	VLPs	Montanide ISA 71 VG	Chicken, IM	Produced high‐titer neutralizing antibodies and virus‐specific IgG	Complete protection against lethal H5N1 and H7N9 challenges	[55]
	HA, M1 (H9N2)	VLPs	Montanide ISA 70	Chicken, IM	Elicited robust immune response	Robust protective immune response; allowed for DIVA strategy	[56]
	HA, M2e	Ferritin nanoparticles	CpG IAMA‐002	Mouse, IN	Elicited strong humoral, cellular and mucosal immune responses	Cross‐protection against homo‐ and heterologous influenza viruses (contain H1N1, H3N2, H5N8, and H9N2)	[57]
	H5 HA	Ferritin nanoparticles	Montanide ISA 78 VG	Chicken, SQ	Elicited potent HI and neutralizing antibodies	100% survival against lethal H5N6 challenge; significantly reduced lung damage	[58]
	H5 HA	I53	Al(OH)_3_	Mouse, IM	Significantly improved antibody levels	Not assessed	[59]
	M2e	Porcine circovirus type 2 VLPs	Thiolated chitosan	Mouse, IN	Significantly enhances M2e‐specific humoral and mucosal immunity, as well as NP epitope‐specific T‐cell immunity	Broad protection against divergent influenza A viruses (H1N1, H3N2, and H9N2)	[60]
	Inactivated H9N2 virus	PLGA	CpG 2007	Chicken, IM	Increased systemic and mucosal IgY levels and HI titers	Significantly reduced viral shedding	[61]
	Inactivated H9N2 virus	PLGA	CpG 2007	Chicken, IM, and aerosol	Significant effects on humoral and mucosal immune pathways	Significantly reduced viral shedding	[62]
	Inactivated virus (mosaic H5)	Polyanhydride	None	Chicken, IM	Induced broad‐spectrum immunity	Significantly reduced viral shedding after heterologous H5N1/H5N2 challenge	[63]
	Trimer of H5 HA	Polyanhydride	poly I:C	Mouse, IM	Induced high levels of neutralizing antibodies and CD4^+^ T‐cell responses	Complete protection against low‐pathogenicity H5N1	[64]
	Inactivated H9N2 virus	Chitosan	Hemokinin‐1	Mouse, ocular	Hemokinin‐1 synergistically improved antibody levels	Not assessed	[65]
	DNA (M1, HA, GM‐CSF)	Chitosan	None	Chicken, IN	Increased antibody titers, activated CD4^+^/CD8^+^ T‐cells, stimulated IL‐4 and IFN‐γ	Reduced pulmonary viral loads and shedding after challenge	[66]
	H5 HA mRNA	Liposome	None	Mouse and ferrets, IM	Induced high‐titer neutralizing and broad‐spectrum antibodies	Significantly reduced morbidity and mortality	[67]
	H5 HA mRNA (self‐amplifying)	Liposome	None	Mouse, IM	Enhanced humoral and cellular immunity	Not assessed	[68]
	H9 HA mRNA	Liposome	None	Chicken, IM	Induced higher specific antibody titers and IFN‐γ expression	Multiorgan viral load was reduced and no pathological changes were observed in the lungs	[69]
	H7 HA	Silicon dioxide	TLR7/8 agonists	Mouse, IM	Enhanced humoral and Th1/Th17‐polarized T cell immune responses	Not assessed	[70]
	M2e	Gold	CpG 1826	Mouse, IN	Triggered strong B‐cell activation and increased IgG	Complete or near‐complete protection against H1N1 (100%), H3N2 (92%), and H5N1 (100%)	[71]
	Inactivated H9N2 virus	Fe_2_O_3_	Carboxymethyl chitosan	Chicken, IM	Enhanced humoral and cellular (Th1) immune responses	No virus shedding detected after challenge	[72]
	Inactivated H5N1 virus	Calcium carbonate	Lentinan	Mouse, SQ	Improved DC maturation, balanced CD4^+^/CD8^+^ T‐cells, high HI titers	Induced stronger cellular and humoral immune responses	[73]

Newcastle disease virus (NDV)	M, F and HN	VLPs	Alum adjuvant	Chicken, IM	Induced strong immune responses	Full protection with reduced virus load and decreased virus shedding	[74]
	F and HN of NDV, M of IAV	VLPs	Montanide ISA 70	Chicken, IM	Dose‐dependent anti‐NDV antibody production	Complete sterilizing protection against lethal challenge; total suppression of viral shedding	[75]
	HA and M of IAV, NA and HN of NDV	VLPs	None	Chicken, SQ	Induced high levels of specific antibodies against AIV H5 and NDV	Complete protection against NDV	[76]
	Inactivated virus	PLGA	None	Chicken, IM	Higher HI antibody titers and IgY levels; increased IL‐4 and IFN‐γ	Complete protection against the NDV	[77]
	Inactivated virus	Chitosan and derivatives	None	Chicken, IM	Significant differences in inducing humoral and cellular immunity	Protective efficacy comparable to commercial vaccines	[78]
	F gene plasmid DNA	Chitosan derivatives (O‐2^′^‐HACC)	None	Chicken, IN	Enhanced humoral, cellular and mucosal immune responses	Protected chickens from virulent NDV infection	[79]
	HN/F gene plasmid DNA	Dextran‐spermine	None	Chicken embryo	Limited antibody response induction	Partial protection against lethal challenge	[80]
	Live attenuated vaccine	Silicon dioxide	PEI	Chicken, IM	Enhanced humoral and cellular immune responses	Full protection against lethal challenge	[81]
	Inactivated virus	Calcium phosphate	None	Chicken, IN	Enhanced mucosal and humoral antibody responses	Partial to complete protection against lethal challenge	[82]

Infectious bronchitis virus (IBV)	S1 (plant‐produced)	VLPs	Emulsigen‐P	Chicken, IM	Elicited S‐specific antibody levels comparable to live vaccines	Significantly reduced viral shedding in trachea and cloaca	[83]
	HR2 of spike protein	SAPNs	None	Chicken, IM	Induced high antibody response	Significant reduction in virus and tracheal lesions	[84]
	IBV RBD	PLGA	CpG 2007	Chicken, SQ	Strong humoral and cellular responses	Increased protection in chickens	[85]
	Inactivated virus	Chitosan	None	Chicken, oculo‐nasal	Enhanced mucosal IgA and IFN‐γ responses	Protection against infection at local and systemic sites	[86]
	Plasmid encoding S1 protein	Chitosan	Saponin	Chicken, IM	Elicited a strong immune response	Protected against infection with both M41 and CR88 IBV strains	[87]
	S	Gold	Freund’s adjuvant	Chicken, IM	Enhanced antibody and T‐cell responses	Superior antiviral protection with reduced symptoms	[88]

Fowl adenovirus (FAdV)	Hexon capsid protein	VLPs of hepatitis B virus	Montanide ISA 71 VG	Chicken, IM	Induced high‐level antibodies	90% protection rate against FAdV‐4	[89]

Marek’s disease virus (MDV)	Infectious BAC20 clone	Chitosan, calcium phosphate	None	Chicken, IM	Sustained antibody response after challenge	Partial protection dependent on virus reconstitution	[90]
	gB, pp38	Liposome	None	Chicken, IM	Activates innate and adaptive immune responses, inducing an antiviral state	Not assessed	[91]

Infectious bursal disease virus (IBDV)	VP2	VLPs	Oil emulsion	Chicken, IM	Induced high titers of antibodies	Complete clinical protection; prevented bursal atrophy	[92, 93]
	VP2	PLGA	*Amomum longiligulare* polysaccharide 1	Chicken, IM	Enhanced antibody and cytokine responses	Not assessed	[94]

Abbreviations: IM, intramuscular; IN, intranasal; RBD, receptor binding domain; SQ, subcutaneous.

### 4.1. VLPs

VLPs are nanostructures that replicate the structure of native viruses but lack genetic material, which contributes to their high safety profiles. They are effective in eliciting strong immune responses by presenting essential viral antigens [95]. Due to these characteristics, VLPs have become a significant area of focus in the research of vaccines for chickens.

AIV VLPs vaccines have shown strong immunoprotective efficacy [96, 97]. The baculovirus/insect cell expression system allows for the efficient production of VLPs that include hemagglutinin (HA), neuraminidase (NA), and matrix (M1) proteins. These VLPs structurally resemble native virions, which range from 80 to 150 nm in diameter, while ensuring biosafety and strong immunogenicity [53, 98]. When a single dose of the bivalent H5 + H7 VLP vaccine was administered to chickens, it resulted in the production of high‐titer neutralizing antibodies and virus‐specific IgG, providing complete protection against lethal challenges from highly pathogenic H5N1 and H7N9 strains. Additionally, this immunization significantly reduced viral shedding and pulmonary pathology [55]. For H9N2‐targeted VLPs, the ISA70‐adjuvanted formulation improved vaccine efficacy and enabled DIVA through a nucleoprotein‐specific ELISA, providing a precise tool for epidemiological monitoring [56]. The H7N9 VLPs activated a balanced Th1/Th2 cellular immune response in both chicken and mouse models, characterized by increased production of interleukin (IL)‐2, IL‐4, and IFN‐γ, along with a reduction in pro‐inflammatory cytokines such as IL‐6 and TNF‐α. This response helped mitigate lung injury and demonstrated cross‐protective efficacy against strains that had undergone antigenic drift [54]. This platform eliminates the reliance on egg‐based manufacturing systems and allows for rapid antigenic adaptation to emerging variants, providing developing countries with a significant strategic advantage for economically viable avian influenza control [99]. Future development priorities will focus on engineering broad‐spectrum multivalent vaccines targeting cocirculating lineages, optimizing combinations of adjuvants and administration routes, and accelerating the transition from preclinical evaluations to clinical applications, ultimately establishing a flexible prevention framework for coordinated control of influenza in both humans and animals [100–102].

NDV VLPs, produced through baculovirus‐mediated coexpression of the fusion (F), HA‐HN, and matrix (M) structural proteins in insect cells, effectively mimic the architecture of native virions and provoke strong immune responses [74, 103]. In chicken models, a single immunization with either 10 or 50 µg of NDV VLPs provided complete sterilizing protection against lethal NDV challenges, resulting in total suppression of viral shedding and allowing for the use of hemagglutination inhibition (HI) assay‐based DIVA strategies to differentiate between infected and vaccinated animals [75]. Mechanistic investigations reveal that NDV VLPs stimulate the TLR4/NF‐κB signaling pathway in DCs, leading to the upregulation of MHC II and costimulatory molecules, which enhances DCs migration and primes CD4^+^ T cells. This dual mechanism also bolsters CD8^+^ T cell‐mediated immunity against virulent strains, achieving a protective efficacy that surpasses traditional live‐vaccine prime‐boost regimens [104]. Additionally, these VLPs can serve as versatile platforms for multivalent vaccine design, as demonstrated by the successful incorporation of the Nipah virus G protein extracellular domain [105, 106]. In the context of combination vaccine development, chimeric VLPs that coexpress AIV components (H5N1 HA and M1) alongside NDV F/HN proteins have been shown to induce HI antibody titers comparable to those of commercial monovalent vaccines after a single‐dose immunization, providing complete protection against NDV challenges while facilitating dual DIVA monitoring through nucleoprotein‐ELISA and HI assays [76, 107]. Furthermore, plant‐based production systems present additional benefits for the manufacturing of thermostable, low‐cost VLPs, which is particularly advantageous for resource‐limited regions [108, 109]. Collectively, these advancements underscore the transformative potential of NDV VLPs and their combinatorial formulations in the development of next‐generation vaccines that offer improved efficacy, safety profiles, and adaptability to epidemiological challenges [95, 110].

Plant‐produced IBV spike (S) protein VLPs, generated through transient expression systems in *Nicotiana benthamiana*, show a remarkable ability to quickly adapt antigenically to emerging variants [109]. These VLPs elicit S‐specific antibody levels that are comparable to those produced by live‐attenuated vaccines, while significantly reducing viral shedding in both the trachea and cloaca after challenge [83]. The advantages of these plant‐derived IBV VLP vaccines include enhanced biosafety, cost‐effectiveness, and the ability to manufacture without eggs, making them a flexible solution for addressing the rapidly changing landscape of IBV genotypes. Additionally, chimeric VLP constructs that combine the M1 protein from H5N1 AIV with IBV S1 proteins have been shown to induce higher levels of neutralizing antibodies and IL‐4 responses in both mouse and chicken models, highlighting their improved immunogenic potential [111, 112].

The development of IBDV vaccines utilizes VP2 self‐assembling VLPs that specifically target new variant strains. Researchs have shown that VP2 VLPs expressed in *E. coli* provided complete clinical protection in chickens, effectively preventing bursal atrophy and offering cross‐protection against classical virulent IBDV [92, 93, 113]. Additionally, VP2 VLPs produced using baculovirus, which were optimized through precursor protein autocleavage engineering, demonstrated the ability to induce a level of humoral immunity comparable to that of commercial inactivated vaccines [114]. This innovative platform supports the DIVA strategy by enabling antibody profiling and validating the bursa‐to‐body weight ratio, thereby establishing a comprehensive approach to managing IBDV variants and coinfections.

Nevertheless, there are ongoing technical challenges in developing VLPs for large‐genome DNA viruses, like FAdV and MDV, and there have been relatively few studies conducted on this topic so far. The technical and economic hurdles associated with producing complex VLPs for certain pathogens highlight a key niche for alternative platforms. For instance, the epitope‐focused design of SAPNs offers a more streamlined production path for targeting conserved viral regions, while biodegradable polymeric nanoparticles provide a versatile and often more cost‐effective “cargo” solution for delivering protein subunits or DNA antigens that are difficult to assemble into VLPs.

### 4.2. SAPNs

Building on the structural mimicry concept of VLPs, SAPNs offer a more modular and design‐focused approach. SAPN‐based vaccines show great promise in preventing infectious diseases in chickens by closely resembling natural viral structures or incorporating essential antigenic epitopes, which significantly boosts their immunogenicity and protective effectiveness. Unlike VLPs that often require the expression of multiple structural proteins, SAPNs can be engineered from a single protein subunit, simplifying production while maintaining the repetitive antigen display crucial for B‐cell activation. These vaccines are becoming a vital alternative to traditional methods due to their enhanced immunogenic strength, favorable safety profile, quick adaptability to emerging viral variants, and efficient production through cost‐effective methods like plant‐based platforms and bacterial expression systems [59, 60, 115]. Their distinctive ability to provide broad‐spectrum protection, stimulate mucosal immunity, and present multiple antigens offers a comprehensive approach to managing diseases such as AIV, IBV, FADV, and IBDV [116, 117].

The ferritin‐based antigen display platform has been effectively utilized to create H5N6 HA self‐assembling nanoparticle vaccines. A single immunization with these vaccines resulted in the production of strong HI and neutralizing antibodies, leading to a remarkable 100% survival rate against lethal viral challenges while significantly reducing lung damage [58]. Additionally, multiepitope nanoparticles, such as CHM‐f, enhance mucosal immunity through intranasal delivery. These nanoparticles incorporate conserved B‐cell and T‐cell epitopes, including HA, M2e, and NP protein, which provide cross‐subtype protection against various influenza strains, including H1N1 and H5N8 [57]. Furthermore, nanoparticles produced by bacteria, such as MsDps2, ferritin, and encapsulin, that display conserved regions of the HA stalk, offer both homologous and heterologous protection in preclinical models, paving the way for the development of a universal influenza vaccine [118].

The SAPNs platform, which displays the HR2 epitope of the IBV spike protein, shows significant promise when combined with the flagellin adjuvant, as it notably reduces tracheal viral shedding and lesion scores while also inducing strong antibody and cellular immune responses [84]. In another study, hepatitis B core protein‐based VLPs that incorporate highly conserved hexon epitopes from FAdV4 have demonstrated an impressive 90% protection rate in chickens, outperforming traditional subunit vaccines and confirming the immunogenicity‐enhancing benefits of nanostructured designs [89]. This innovative approach takes advantage of the structural conservation found in adenovirus hexon epitopes, paving the way for new strategies in pan‐adenovirus vaccine development. Additionally, chimeric IBDV capsids, such as HT‐VP2‐466, have facilitated the incorporation of heterologous antigens, including the influenza HA stem and M2 epitopes, resulting in multifunctional vaccine platforms that provided complete protection in mouse models [119]. This modular design strategy represents a significant advancement in multivalent vaccine approaches aimed at combating cocirculating pathogens in chickens. This modularity positions SAPNs between the native structure presentation of VLPs and the antigen encapsulation approaches of polymeric nanoparticles, offering a unique balance of design precision and immunological potency.

### 4.3. Biodegradable Polymeric Nanoparticles

Diverging from the preassembled nanostructures of VLPs and SAPNs, biodegradable polymeric nanoparticles represent a distinct strategy centered on controlled antigen release and codelivery. These nanoparticles have emerged as a promising platform for vaccine delivery, providing a controlled release of antigens that extends the exposure of immunogens and enhances the persistence of the immune response [120, 121]. This “depot effect” stands in contrast to the immediate antigen availability offered by VLPs, instead leveraging prolonged stimulation to bolster T‐cell immunity and memory responses. Additionally, these nanoparticles can codeliver immunostimulants, which further boosts the efficacy of vaccines. Recent advancements in this area have shown considerable potential for improving protective immunity against avian influenza and other infectious diseases in chickens [62, 77, 122, 123].

Studies have shown that poly(lactic‐co‐glycolic acid) (PLGA) nanoparticles encapsulating the TLR21 agonist CpG ODN 2007 significantly boost vaccine immunogenicity [62]. When the unencapsulated H9N2 inactivated virus was administered intramuscularly alongside PLGA–CpG nanoparticles, there was a notable increase in systemic and mucosal IgY antibody levels, improved HI titers, and enhanced antibody avidity through TLR21 signaling, which led to a significant reduction in viral shedding following challenge [61]. In the context of Newcastle disease vaccine development, PLGA‐encapsulated inactivated NDV resulted in higher HI antibody titers and IgY levels compared to traditional oil‐adjuvanted vaccines, along with increased expression of IL‐4 and IFN‐γ, indicating a balanced Th1/Th2 immune response [77]. Furthermore, PLGA‐encapsulated DNA vaccines, such as pVAX1‐F that expresses the NDV F gene, demonstrated a 93% sustained release of DNA, leading to enhanced cellular, humoral, and mucosal immunity, ultimately resulting in complete protection against the virus. This capability to deliver genetic material contrasts with the protein‐only payload of most VLPs and SAPNs, and offers a different mechanism of action compared to the mRNA delivery of LNPs, typically resulting in more prolonged but lower‐level antigen expression. The sustained release kinetics and targeted delivery capabilities of PLGA are key factors in its ability to enhance immunogenicity [124]. In the case of infectious bronchitis, the combination of subunit vaccines with hollow PLGA nanoparticles significantly improved humoral immunity and antiviral protection [85]. Additionally, research on IBDV showed that ALP1–VP2–PLGA nanoparticles not only improved the bursa‐to‐body weight ratios but also increased VP2‐specific antibodies and stimulated the production of proinflammatory cytokines, IL‐2 and TNF‐α, highlighting their dual role in enhancing immunity and promoting growth [94].

Polyanhydride nanoparticle‐encapsulated recombinant H5 trimer antigens have been shown to elicit strong immune responses, including high‐titer neutralizing antibodies and CD4^+^ T‐cell responses, in mouse models, providing protection against low‐pathogenicity H5N1 challenges [64]. Additionally, a mosaic H5 vaccine, which was delivered using sustained‐release polyanhydride nanoparticles or modified vaccinia Ankara vectors, demonstrated the ability to induce broad‐spectrum immunity in chickens. This approach significantly reduced viral shedding after challenges with heterologous H5N1 and H5N2 strains while also ensuring the maintenance of durable humoral and cellular memory [63]. These innovative platforms address the limitations of traditional vaccines by enhancing antigen persistence and enabling cross‐protective mechanisms, thus presenting essential technological strategies for pandemic preparedness and the control of multisubtype avian influenza.

### 4.4. Polysaccharide‐Based Nanoparticles

While many platforms focus on systemic immunity, polysaccharide‐based nanoparticles, particularly those derived from chitosan, specialize in enhancing mucosal immunity. Mucosal immunity is a critical first line of defense for respiratory pathogens. These nanoparticles have become crucial in vaccine carrier research because of their outstanding biocompatibility, biodegradability, and intrinsic mucoadhesive properties. These nanostructures are effective at encapsulating vaccine antigens and play a significant role in activating immune responses by interacting specifically with immune cells [125, 126]. For instance, certain nanoparticles made from polysaccharides can boost the activation of macrophages and DCs, which enhances their ability to present antigens and stimulate strong responses from T‐cells and B‐cells [127–129]. These immunostimulatory properties are fundamental to their effectiveness in vaccines for chickens, providing innovative approaches to tackle viral pathogens affecting chicken.

Chitosan and its derivatives play important roles as both adjuvants and delivery vehicles in chicken viral vaccines, significantly improving mucosal immunity, cellular responses, and the stability of antigens [130, 131]. Their natural bioadhesion provides a distinct advantage over synthetic polymeric nanoparticles for intranasal and oral delivery, promoting longer residence time at mucosal surfaces and enhancing antigen uptake by mucosal‐associated lymphoid tissue. In the development of the H9N2 vaccine, chitosan acts as a natural adjuvant and carrier. For instance, chitosan‐encapsulated DNA vaccines, like the pYL233 construct that encodes the M1 and HA proteins, create nanoparticles with an average diameter of 150 nm, achieving an impressive encapsulation efficiency of 93.2%. This formulation effectively protects the DNA from degradation by nucleases while ensuring stability at 37°C. When administered intranasally as a booster, this nanovaccine significantly increased antibody titers, activated CD4^+^ and CD8^+^ T‐cells, stimulated lymphocyte proliferation, and enhanced the secretion of IL‐4 and IFN‐γ in chickens. Additionally, it led to a reduction in pulmonary viral loads and shedding after the challenge [66]. The combined use of chitosan nanoparticles with the molecular adjuvant HK‐1 resulted in higher and more sustained antibody titers, showcasing chitosan’s ability to enhance systemic immunity through collaborative adjuvant effects [65].

Chitosan derivatives, such as hydroxypropyltrimethyl ammonium chloride chitosan (HACC), sulfated chitosan (SCS), and O‐2^′^‐HACC, have shown remarkable mucosal immunoenhancing properties in NDV vaccines. For instance, HACC/chitosan nanoparticles, which have a particle size of 156.2 nm, have been found to promote superior cellular immunity, as evidenced by increased CD4^+^/CD8^+^ T‐cell ratios and enhanced cytokine secretion. Notably, these nanoparticles exhibit protective efficacy against highly pathogenic NDV that is comparable to that of commercial oil‐emulsion vaccines, even though they induce lower humoral immune responses [78]. Additionally, O‐2^′^‐HACC‐encapsulated NDV F gene plasmids, when administered intranasally, triggered higher levels of IgG, secretory IgA, and mucosal immunity compared to traditional intramuscular vaccines [79]. In contrast, other polysaccharides, such as dextran‐spermine, have shown less effective DNA delivery capabilities, with unencapsulated DNA vaccines performing better than their nanoparticle counterparts [80].

Chitosan nanoparticles play a crucial role in enhancing the effectiveness of both inactivated and DNA vaccines against IBV. Inactivated IBV vaccines encapsulated in chitosan administered via ocular‐nasal routes significantly boosted mucosal immunity through elevated IgA and IFN‐γ production while suppressing viral replication in tracheal and renal tissues [86]. Additionally, a bivalent S1 glycoprotein DNA vaccine targeting M41 and CR88 strains, delivered in chitosan‐saponin nanoparticles, induced potent humoral immunity and cellular responses, markedly reducing viral shedding and histopathological lesions without requiring multiple boosters [87].

Chitosan nanoparticles were explored as delivery vehicles for an MDV DNA vaccine known as BAC20. However, their protective efficacy was found to be limited, and experimental findings indicated that vaccination with chitosan‐encapsulated BAC20 DNA only postponed the onset of the disease. In contrast, viral reconstitution‐based vaccines, such as CV1988, demonstrated significantly higher protection rates, with five out of seven subjects surviving. This suggests that in vivo viral reconstitution plays a crucial role in developing protective immunity [90].

### 4.5. LNPs

Representing the cutting‐edge of nucleic acid delivery, LNPs share the encapsulation strategy of polymeric nanoparticles but are uniquely optimized for the efficient delivery of fragile RNA payloads. LNPs act as versatile carriers for vaccines by encapsulating both hydrophilic and lipophilic antigens, which improves their stability and bioavailability while also promoting both humoral and cellular immune responses [132]. Their rapid development cycle and potent immunogenicity position them differently from the protein‐based VLPs and SAPNs, offering unparalleled speed in response to emerging viral variants. When combined with mRNA technology, known as mRNA–LNPs, these carriers allow for quick adjustments to address viral variants by eliciting dual responses from antibodies and T cells [133]. Additionally, their compatibility with lyophilization makes them suitable for use in tropical regions [134, 135]. However, significant challenges persist, including the need for optimized delivery methods, cost‐effective scalability, and consistent manufacturing processes to ensure successful global implementation [116, 136].

An mRNA–LNP vaccine targeting H5‐subtype AIVs, especially those from the 2.3.4.4b clade, has shown promising results by encoding the HA protein, which led to the production of high‐titer neutralizing antibodies and broad‐spectrum anti‐HA stalk antibodies in both mice and ferret models. This vaccine significantly reduced morbidity and mortality following viral challenges [67]. Additionally, self‐amplifying mRNA designs that included membrane‐anchored full‐length HA were found to enhance both humoral and cellular immunity, resulting in pulmonary IgA responses and strong activation of CD4^+^ and CD8^+^ T‐cells [68]. Chicken model evaluations confirmed the safety profile of mRNA‐LNPs vaccines, with no clinical pathology observed, coupled with robust humoral immunity evidenced by elevated HI antibody titers and cellular immunity characterized by increased IFN‐γ secretion, resulting in substantial viral load reduction across multiple organs [69]. For MDV, mRNA vaccines encoding gB/pp38 proteins, administered in a two‐dose regimen, were effective in activating innate immune pathways through the upregulation of IFN‐α/β, MDA5, and Mx1, while also stimulating adaptive immunity by elevating IL‐2 levels, which effectively suppressed viral replication [91]. Collectively, mRNA–LNPs vaccines present a strategic approach to combat viral threats in chickens due to their rapid development, strong immunogenicity, and favorable safety profiles [137, 138]. However, their current high cost and cold‐chain requirements present distinct practical challenges compared to more stable platforms like inorganic nanoparticles or some polymeric formulations, highlighting the trade‐off between technological sophistication and field deployability.

### 4.6. Inorganic Nanoparticles

Completing the spectrum of nanoplatforms, inorganic nanoparticles fulfill a specialized role as potent immune enhancers and versatile scaffolds, often with inherent adjuvant properties not found in organic materials. They have garnered considerable interest for their application in chicken vaccines, serving both as carriers and immune enhancers. Silica nanoparticles, in particular, exhibit multifunctional capabilities in vaccine delivery [139]. For instance, amine‐modified silica nanoparticles that codeliver TLR7/8 agonists alongside AIV H7 antigens have been shown to significantly boost the activation of APCs and maintain Th1/Th17‐polarized immune responses through extended immunostimulation. Research has demonstrated that the size of silica nanoparticles, ranging from 50 to 200 nm, as well as the density of surface ligands, play crucial roles in determining their immunogenicity. Notably, smaller particles, specifically those measuring 50 nm, and those with lower ligand density, exhibited superior efficacy [70]. Additionally, biomimetic silicified nanoparticles, when optimized for NDV attenuated vaccines, have shown to enhance antibody persistence and promote CD3^+^CD4^+^ T‐cell proliferation, ultimately achieving complete protection following a challenge. Furthermore, polyethyleneimine‐modified silica nanoparticles have been found to improve the mucosal delivery of NDV live‐attenuated vaccines [81].

Gold nanoparticles serve as versatile carriers for broad‐spectrum influenza vaccines [140–142]. When M2e peptide‐CpG complexes are delivered intranasally, they trigger strong activation of B‐cells in the lungs and lead to significantly increased levels of IgG antibodies. This response results in complete or near‐complete protection against various influenza strains, including H1N1 (100%), H3N2 (92%), and H5N1 (100%) [71]. Additionally, synthetic VLPs that are designed with spike protein coronas from the IBV closely resemble natural viruses. This resemblance improves the delivery of antigens to the lymphatic system, boosts antibody production, and enhances T‐cell responses, making them more effective than conventional inactivated vaccines [88].

Iron oxide nanoparticles functionalized with carboxymethyl chitosan acted as adjuvants when combined with irradiated H9N2 antigens, boosting HI antibody titers and Th1‐type immunity without activating Th2 pathways, thus favoring cell‐mediated protection [72]. Similarly, calcium carbonate–lentinan (CaCO_3_–LNT) composite microparticles (2 μm), have been shown to improve DC maturation, achieve a balanced ratio of CD4^+^–CD8^+^ T‐cells, and stimulate high HI titers along with Th‐associated cytokines, thereby creating a synergistic effect between humoral and cellular immunity [73]. In contrast, calcium phosphate adjuvants used in NDV inactivated vaccines demonstrated a weaker systemic immune response compared to chitosan but still maintained a modest potential for mucosal immunostimulation [82, 143].

Inorganic nanoparticles offer adjustable size, surface modifications, and controlled release mechanisms, making them valuable in various applications for chicken vaccines. For instance, silica and gold nanoparticles are particularly effective in delivering antigens alongside adjuvants, which can enhance cross‐protection against diseases [144]. On the other hand, iron oxide and calcium carbonate nanoparticles play a crucial role in boosting cellular immunity [145–148]. While many of these inorganic nanoparticles show promise in improving immune responses, it is essential to conduct thorough assessments of their long‐term safety. The potential for persistence that raises safety concerns for nondegradable inorganic nanoparticles is precisely the attribute that distinguishes them from biodegradable polymers and contributes to their prolonged immunostimulatory effect. This fundamental trade‐off between durability and safety underscores the importance of matching platform selection to specific application needs. This is necessary to ensure that they enhance vaccine effectiveness without negatively impacting the health of chickens. Moving forward, research should focus on improving biocompatibility and targeting precision, striking a balance between safety and efficacy. This approach will help advance the use of these materials in practical applications within the field of chicken vaccinology [149–152].

The distinct profiles of these nanovaccine platforms present a spectrum of options for vaccine design. Their key characteristics are systematically compared in Table 3, highlighting that the optimal choice is contingent on the specific pathogen, the desired immune response, and overarching economic constraints.

**Table 3:** Comparative analysis of key nanovaccine platforms for chicken applications.

Nanoplatform	Key advantages	Typical immune profile	Scalability and cost (relative)	Key challenges
VLPs	Authentic antigen presentation and high safety	Strong humoral immunity	Medium‐to‐low/high	Complex; costly production for large‐genome viruses
SAPNs	Precise epitope display and high stability	Humoral and cellular immunity	Medium/medium	Designing immunodominant epitopes
Polymeric nanoparticles	Controlled release, co‐delivery	Balanced Th1/Th2; strong cellular	High/low‐to‐medium	Burst release; acidic degradation
Polysaccharide nanoparticles	Mucosal delivery and biocompatibility	Mucosal and systemic immunity	High/low	Variable efficacy based on derivative
LNPs	mRNA/protein delivery and potent activation	Potent humoral and cellular immunity	Medium/high	Cost; Long‐term safety data
Inorganic nanoparticles	Tunable adjuvantity and stability	Enhanced APC activation	High/low	Long‐term biodistribution and safety

## 5. Challenges and Future Directions

Despite their considerable promise, the translation of nanoparticle‐based vaccines from laboratory concepts to widely adopted chicken health tools faces a complex set of interconnected challenges.

### 5.1. Scientific Hurdles in Immunogenicity and Safety

A deeper understanding of the variable immunogenicity across different nanoplatforms is needed. While VLPs and SAPNs are adept at inducing high‐titer neutralizing antibodies, their efficacy can be influenced by subtle differences in assembly fidelity and epitope density [153]. Conversely, polymeric and LNPs often promote stronger T‐cell immunity through sustained antigen release, but this very mechanism can sometimes lead to unpredictable reactogenicity [154]. The long‐term biological fate and biocompatibility of these materials present another critical frontier. Biodegradable polymers like PLGA require precise tuning of their degradation kinetics to avoid inflammatory microenvironments that could undermine vaccine efficacy [155]. For nondegradable inorganic nanoparticles, comprehensive studies on their biodistribution and potential for organ‐specific accumulation in chickens are a mandatory prerequisite for ensuring animal safety and food security [155, 156].

### 5.2. Practical and Economic Considerations for Chicken Nanovaccines

The commercial viability of these advanced vaccines is perhaps the most significant barrier to their adoption. The chicken industry operates with strict cost constraints, where a vaccine dose must typically remain below USD 0.10 to be feasible. Current manufacturing processes for many sophisticated platforms, including those using PLGA, LNPs, and complex VLPs, far exceed this threshold due to expensive raw materials and specialized production requirements. Bridging this economic gap demands a paradigm shift towards scalable and low‐cost production from the outset. Promising paths include leveraging plant‐based expression systems for viral proteins, optimizing bacterial fermentation for protein nanoparticles, and designing simpler nanocarriers that forgo complex functionalization [157, 158]. Furthermore, the development of thermostable formulations through techniques like lyophilization is essential to reduce dependence on costly cold‐chain logistics, particularly in resource‐limited settings [159].

### 5.3. Navigating Regulatory and Ethical Landscapes

The regulatory pathway for veterinary nanovaccines remains ambiguous, as existing guidelines were not designed for the unique properties of nanoscale formulations. Proactive collaboration between developers and regulatory agencies is urgently needed to establish standardized characterization protocols for critical quality attributes such as particle size, stability, and sterility [117, 160]. This journey also demands a proactive ethical framework guided by a One Health perspective [161]. This necessitates rigorous assessment of potential nanocarrier residues in edible tissues and eggs to guarantee food safety, while also incorporating animal welfare considerations through careful monitoring for adverse reactions and adherence to the “3Rs” principle (Replacement, Reduction, and Refinement) in challenge trials [162, 163]. Furthermore, the environmental ethics of large‐scale use require thorough ecotoxicological studies to evaluate the ecosystem‐level consequences of excreted nanoparticles on soil health, water quality, and nontarget organisms. Ultimately, conducting large‐scale field trials under diverse commercial conditions is the critical step to validate laboratory efficacy and build confidence among end‐users and regulators [164].

## 6. Conclusion and Future Perspectives

Nanoparticle‐based vaccines represent a transformative advancement in the quest to combat economically devastating viral diseases in chickens. By enabling precise antigen delivery and enhanced immune activation, these platforms directly address the core limitations of conventional vaccines, namely their limited cross‐protection and durability. A comparative view reveals a versatile toolkit where each platform offers distinct advantages. VLPs and SAPNs mimic natural viruses to drive potent antibody responses. Lipid and polymeric nanoparticles act as versatile cargo ships to promote sustained T‐cell immunity. Inorganic particles provide potent adjuvant effects. However, their practical value is balanced against scalability and cost, creating a clear trade‐off between technological sophistication and field deployability.

To realize the full potential of this technology and translate it into widespread practice, future efforts must strategically address the critical gaps identified in this review. Scientifically, a deeper mechanistic understanding of cross protection is paramount to guide the rational design of broad spectrum vaccines targeting conserved viral epitopes. Technologically, platform specific challenges must be overcome, including streamlining the complex production of VLPs for certain pathogens, optimizing the biocompatibility and controlled release of biodegradable polymers, and conducting thorough safety assessments of non degradable nanoparticles in food animals. The concurrent development of affordable and thermostable formulations is essential to enhance global accessibility and reduce cold chain reliance. Practically, the establishment of universal standardization protocols for critical quality attributes forms the foundation for regulatory confidence and must be coupled with robust efficacy data from large scale field trials. Success in this endeavor will hinge on sustained multidisciplinary collaboration that integrates pathogen biology, immunology, and materials science with industrial manufacturing and regulatory science. By focusing on these strategic priorities, nanoparticle vaccines can evolve from promising prototypes into practical and powerful tools for safeguarding global chicken health.

## Disclosure

All the authors contributed to the final article revision and approved the submitted version.

## Conflicts of Interest

The authors declare no conflicts of interest.

## Author Contributions

Peiyang Ding contributed to conception, project administration, manuscript revision, and funding support. Peiyang Ding, Litong Xia, and Shuoqi Dong wrote and revised the original manuscript. Peiyang Dong and Litong Xia prepared the figures.

## Funding

This work was funded by the China Postdoctoral Science Foundation (Grant 2023M743209) and the Key R and D and Promotion Projects in Henan Province of China (Grant 252102111011).

## Data Availability

The data sharing is not applicable to this article as no datasets were generated or analyzed during the current study.

## References

[1] Li H. , Liu G. , and Zhou Q. , et al.Which Strain of the Avian Coronavirus Vaccine Will Become the Prevalent One in China Next?, Frontiers in Veterinary Science. (2023) 10, 10.3389/fvets.2023.1139089, 1139089. 37215473 PMC10196085

[2] Dey P. , Ahuja A. , and Panwar J. , et al.Immune Control of Avian Influenza Virus Infection and Its Vaccine Development, Vaccines. (2023) 11, no. 3, 10.3390/vaccines11030593. PMC10058720 36992177

[3] Hu Z. , Ai H. , and Wang Z. , et al.Impact of Inactivated Vaccine on Transmission and Evolution of H9N2 Avian Influenza Virus in Chickens, npj Vaccines. (2025) 10, no. 1, 10.1038/s41541-025-01115-y. PMC11971428 40185759

[4] Wang T. , Wei F. , and Liu J. , Emerging Role of Mucosal Vaccine in Preventing Infection With Avian Influenza A Viruses, Viruses. (2020) 12, no. 8, 10.3390/v12080862, 862. 32784697 PMC7472103

[5] Kanaujia R. , Bora I. , Ratho R. K. , Thakur V. , Mohi G. K. , and Thakur P. , Avian Influenza Revisited: Concerns and Constraints, VirusDisease. (2022) 33, no. 4, 456–465, 10.1007/s13337-022-00800-z. 36320191 PMC9614751

[6] Faccin F. C. and Perez D. R. , Pandemic Preparedness Through Vaccine Development for Avian Influenza Viruses, Human Vaccines & Immunotherapeutics. (2024) 20, no. 1. 10.1080/21645515.2024.2347019 PMC11141480 38807261

[7] Hu Z. , He X. , Deng J. , Hu J. , and Liu X. , Current Situation and Future Direction of Newcastle Disease Vaccines, Veterinary Research. (2022) 53, no. 1, 10.1186/s13567-022-01118-w, 99. 36435802 PMC9701384

[8] Legnardi M. , Tucciarone C. M. , Franzo G. , and Cecchinato M. , Infectious Bronchitis Virus Evolution, Diagnosis and Control, Veterinary Sciences. (2020) 7, no. 2, 10.3390/vetsci7020079, 79. 32580381 PMC7356646

[9] Shah M. S. , Ashraf A. , and Khan M. I. , et al.Fowl Adenovirus: History, Emergence, Biology and Development of a Vaccine Against Hydropericardium Syndrome, Archives of Virology. (2017) 162, no. 7, 1833–1843, 10.1007/s00705-017-3313-5, 2-s2.0-85014760180. 28283816

[10] De Luca C. and Hess M. , Vaccination Strategies to Protect Chickens From Fowl Adenovirus (FAdV)-Induced Diseases: A Comprehensive Review, Vaccine. (2025) 43, no. Pt 1, 10.1016/j.vaccine.2024.126496, 126496. 39522325

[11] Davidson I. , Out of Sight, but Not Out of Mind: Aspects of the Avian Oncogenic Herpesvirus, Marek’s Disease Virus, Animals. (2020) 10, no. 8, 10.3390/ani10081319, 1319. 32751762 PMC7459476

[12] Jackwood D. J. , Advances in Vaccine Research Against Economically Important Viral Diseases of Food Animals: Infectious Bursal Disease Virus, Veterinary Microbiology. (2017) 206, 121–125, 10.1016/j.vetmic.2016.11.022, 2-s2.0-85007495903. 27916318

[13] Liu F. , Wu X. , Li L. , Ge S. , Liu Z. , and Wang Z. , Virus-Like Particles: Promising Platforms With Characteristics of DIVA for Veterinary Vaccine Design, Comparative Immunology, Microbiology and Infectious Diseases. (2013) 36, no. 4, 343–352, 10.1016/j.cimid.2013.02.002, 2-s2.0-84878976780. 23561290

[14] Tabynov K. , Kuanyshbek A. , and Yelchibayeva L. , et al.Evaluation of Safety, Immunogenicity, and Efficacy of Inactivated Reverse-Genetics-Based H5N8 Highly Pathogenic Avian Influenza Virus Vaccine With Various Adjuvants via Parenteral and Mucosal Routes in Chickens, Frontiers in Immunology. (2025) 16, 10.3389/fimmu.2025.1539492, 1539492. 40181968 PMC11965622

[15] Nurzijah I. , Elbohy O. A. , Kanyuka K. , Daly J. M. , and Dunham S. , Development of Plant-Based Vaccines for Prevention of Avian Influenza and Newcastle Disease in Poultry, Vaccines. (2022) 10, no. 3, 10.3390/vaccines10030478, 478. 35335110 PMC8952014

[16] Ganar K. , Das M. , Sinha S. , and Kumar S. , Newcastle Disease Virus: Current Status and Our Understanding, Virus Research. (2014) 184, 71–81, 10.1016/j.virusres.2014.02.016, 2-s2.0-84898621428. 24589707 PMC7127793

[17] Berihulay H. , Luo W. , and Lao A. , et al.Exploring the Genetic Basis of Newcastle Disease Virus in Chickens: A Comprehensive Review, Frontiers in Immunology. (2025) 16, 10.3389/fimmu.2025.1614794, 1614794. 40655137 PMC12245680

[18] Zhang D. , Ding Z. , and Xu X. , Pathologic Mechanisms of the Newcastle Disease Virus, Viruses. (2023) 15, no. 4, 10.3390/v15040864, 864. 37112843 PMC10143668

[19] Bello M. B. , Yusoff K. , Ideris A. , Hair-Bejo M. , Peeters B. P. H. , and Omar A. R. , Diagnostic and Vaccination Approaches for Newcastle Disease Virus in Poultry: The Current and Emerging Perspectives, BioMed Research International. (2018) 2018, 7278459. 30175140 10.1155/2018/7278459 PMC6098882

[20] Hu Z. , Ni J. , Cao Y. , and Liu X. , Newcastle Disease Virus as a Vaccine Vector for 20 Years: A Focus on Maternally Derived Antibody Interference, Vaccines. (2020) 8, no. 2, 10.3390/vaccines8020222, 222. 32422944 PMC7349365

[21] Falchieri M. , Coward V. J. , Reid S. M. , Lewis T. , and Banyard A. C. , Infectious Bronchitis Virus: An Overview of the “Chicken Coronavirus”, Journal of Medical Microbiology. (2024) 73, no. 5, 10.1099/jmm.0.001828. PMC11184965 38771617

[22] Bhuiyan M. S. A. , Amin Z. , and Rodrigues K. F. , et al.Infectious Bronchitis Virus (Gammacoronavirus) in Poultry Farming: Vaccination, Immune Response and Measures for Mitigation., Veterinary sciences. (2021) 8, no. 11, 10.3390/vetsci8110273. PMC8623603 34822646

[23] Zhao J. , Zhao Y. , and Zhang G. , Key Aspects of Coronavirus Avian Infectious Bronchitis Virus, Pathogens. (2023) 12, no. 5, 10.3390/pathogens12050698, 698. 37242368 PMC10221305

[24] Jordan B. , Vaccination Against Infectious Bronchitis Virus: A Continuous Challenge, Veterinary Microbiology. (2017) 206, 137–143, 10.1016/j.vetmic.2017.01.002, 2-s2.0-85021784576. 28081857

[25] Wang Z. and Zhao J. , Pathogenesis of Hypervirulent Fowl Adenovirus Serotype 4: The Contributions of Viral and Host Factors, Viruses. (2019) 11, no. 8, 10.3390/v11080741, 2-s2.0-85071280138, 741. 31408986 PMC6723092

[26] Mo J. , Historical Investigation of Fowl Adenovirus Outbreaks in South Korea From 2007 to 2021: A Comprehensive Review, Viruses. (2021) 13, no. 11, 10.3390/v13112256. PMC8621494 34835062

[27] Schachner A. , Matos M. , Grafl B. , and Hess M. , Fowl Adenovirus-Induced Diseases and Strategies for Their Control—A Review on the Current Global Situation, Avian Pathology. (2018) 47, no. 2, 111–126, 10.1080/03079457.2017.1385724, 2-s2.0-85042494354. 28950714

[28] Kardoudi A. , Benani A. , and Allaoui A. , et al.Fowl Adenovirus Serotype 1: From Gizzard Erosion to Comprehensive Insights into Genome Organization, Epidemiology, Pathogenesis, Diagnosis, and Prevention, Veterinary Sciences. (2025) 12, no. 4, 10.3390/vetsci12040378, 378. 40284880 PMC12030904

[29] Zhu Z. J. , Teng M. , and Liu Y. , et al.Immune Escape of Avian Oncogenic Marek’s Disease Herpesvirus and Antagonistic Host Immune Responses, npj Vaccines. (2024) 9, no. 1, 10.1038/s41541-024-00905-0, 109. 38879650 PMC11180173

[30] Boodhoo N. , Gurung A. , Sharif S. , and Behboudi S. , Marek’s Disease in Chickens: A Review With Focus on Immunology, Veterinary Research. (2016) 47, no. 1, 10.1186/s13567-016-0404-3, 2-s2.0-84997523901, 119. 27894330 PMC5127044

[31] Yang Y. , Dong M. , Hao X. , Qin A. , and Shang S. , Revisiting Cellular Immune Response to Oncogenic Marek’s Disease Virus: The Rising of Avian T-Cell Immunity, Cellular and Molecular Life Sciences. (2020) 77, no. 16, 3103–3116, 10.1007/s00018-020-03477-z. 32080753 PMC7391395

[32] Bertzbach L. D. , Conradie A. M. , You Y. , and Kaufer B. B. , Latest Insights Into Marek’s Disease Virus Pathogenesis and Tumorigenesis, Cancers. (2020) 12, no. 3, 10.3390/cancers12030647, 647. 32164311 PMC7139298

[33] Davison F. and Nair V. , Use of Marek’s Disease Vaccines: Could They be Driving the Virus to Increasing Virulence?, Expert Review of Vaccines. (2014) 4, no. 1, 77–88, 10.1586/14760584.4.1.77, 2-s2.0-14744281511. 15757475

[34] Qin Y. and Zheng S. , Infectious Bursal Disease Virus-Host Interactions: Multifunctional Viral Proteins That Perform Multiple and Differing Jobs, International Journal of Molecular Sciences. (2017) 18, no. 1, 10.3390/ijms18010161, 2-s2.0-85009932255, 161. 28098808 PMC5297794

[35] Yang H. and Ye C. , Reverse Genetics Approaches for Live-Attenuated Vaccine Development of Infectious Bursal Disease Virus, Current Opinion in Virology. (2020) 44, 139–144, 10.1016/j.coviro.2020.08.001. 32892072

[36] Müller H. , Mundt E. , Eterradossi N. , and Islam M. R. , Current Status of Vaccines Against Infectious Bursal Disease, Avian Pathology. (2012) 41, no. 2, 133–139, 10.1080/03079457.2012.661403, 2-s2.0-84860125708. 22515532

[37] Gheibi Hayat S. M. and Darroudi M. , Nanovaccine: A Novel Approach in Immunization, Journal of Cellular Physiology. (2019) 234, no. 8, 12530–12536, 10.1002/jcp.28120, 2-s2.0-85059893013. 30633361 PMC13484040

[38] Pati R. , Shevtsov M. , and Sonawane A. , Nanoparticle Vaccines Against Infectious Diseases, Frontiers in Immunology. (2018) 9, 10.3389/fimmu.2018.02224, 2-s2.0-85055078692, 2224. 30337923 PMC6180194

[39] Bhardwaj P. , Bhatia E. , Sharma S. , Ahamad N. , and Banerjee R. , Advancements in Prophylactic and Therapeutic Nanovaccines, Acta Biomaterialia. (2020) 108, 1–21, 10.1016/j.actbio.2020.03.020. 32268235 PMC7163188

[40] Wang L. , Wang X. Y. , and Yang F. M. , et al.Systemic Antiviral Immunization by Virus-Mimicking Nanoparticles- Decorated Erythrocytes, Nano Today. (2021) 40, 10.1016/j.nantod.2021.101280, 101280. 34512795 PMC8418322

[41] Van Lysebetten D. , Malfanti A. , and Deswarte K. , et al.Lipid-Polyglutamate Nanoparticle Vaccine Platform, ACS Applied Materials & Interfaces. (2021) 13, no. 5, 6011–6022, 10.1021/acsami.0c20607. 33507728 PMC7116839

[42] Liu S. , Hu M. L. , and Liu X. Q. , et al.Nanoparticles and Antiviral Vaccines, Vaccines-Basel. (2024) 12, no. 1.

[43] Fawzy M. , Khairy G. M. , Hesham A. , Rabaan A. A. , El-Shamy A. G. , and Nagy A. , Nanoparticles as a Novel and Promising Antiviral Platform in Veterinary Medicine, Archives of Virology. (2021) 166, no. 10, 2673–2682, 10.1007/s00705-021-05177-w. 34297222 PMC8298697

[44] Pan C. , Yu S. , and Li C. , et al.Rapid and Efficient Immune Response Induced by a Designed Modular Cholera Toxin B Subunit (CTB)-Based Self-Assembling Nanoparticle, Biomaterials. (2025) 315, 10.1016/j.biomaterials.2024.122946, 122946. 39515192

[45] Howard G. P. , Bender N. G. , and Khare P. , et al.Immunopotentiation by Lymph-Node Targeting of a Malaria Transmission-Blocking Nanovaccine, Frontiers in Immunology. (2021) 12, 10.3389/fimmu.2021.729086, 729086. 34512663 PMC8432939

[46] Garg A. , Agrawal R. , Chopra H. , Singh T. , Chaudhary R. , and Tankara A. , A Glance on Nanovaccine: A Potential Approach for Disease Prevention, Current Pharmaceutical Biotechnology. (2024) 25, no. 11, 1406–1418, 10.2174/0113892010254221231006100659. 37861010

[47] Wang X. , Shi G. , and Zhu X. , et al.Engineered Bacteria-Nanoparticle Conjugate Reprograms Immunosuppressive Niche via Dendritic Cell-Centric Innate-Adaptive Immune Coupling, ACS Nano. (2025) 19, no. 27, 24938–24953, 10.1021/acsnano.5c03960. 40603259

[48] Muñoz-Wolf N. and Lavelle E. C. , Promotion of Trained Innate Immunity by Nanoparticles, Seminars in Immunology. (2021) 56, 10.1016/j.smim.2021.101542, 101542. 34973890

[49] Popa A. and Springer S. , Tailored Nanoparticles as Vaccine Components, Applied Sciences. (2021) 11, no. 24, 10.3390/app112411898, 11898.

[50] Zhao L. , Seth A. , and Wibowo N. , et al.Nanoparticle Vaccines, Vaccine. (2014) 32, no. 3, 327–337. 24295808 10.1016/j.vaccine.2013.11.069

[51] Pine M. , Arora G. , and Hart T. M. , et al.Development of an mRNA-Lipid Nanoparticle Vaccine Against Lyme Disease, Molecular Therapy. (2023) 31, no. 9, 2702–2714, 10.1016/j.ymthe.2023.07.022. 37533256 PMC10492027

[52] Patil V. , Renu S. , and Feliciano-Ruiz N. , et al.Intranasal Delivery of Inactivated Influenza Virus and Poly(I:C) Adsorbed Corn-Based Nanoparticle Vaccine Elicited Robust Antigen-Specific Cell-Mediated Immune Responses in Maternal Antibody Positive Nursery Pigs, Frontiers in Immunology. (2020) 11, 10.3389/fimmu.2020.596964, 596964. 33391267 PMC7772411

[53] Luqman M. , Rahman S. U. , Gul S. T. , and Mahmood M. S. , Beyond Traditional Vaccines: Semi-Purified Low-Pathogenic Avian Influenza H9N2 Virus-Like Particles and Their Promise for Broiler Immunity, Veterinary World. (2024) 17, no. 10, 2311–2321, 10.14202/vetworld.2024.2311-2321. 39619921 PMC11606278

[54] Hu J. , Zhang Q. , and Peng P. , et al.Baculovirus-Derived Influenza Virus-Like Particle Confers Complete Protection Against Lethal H7N9 Avian Influenza Virus Challenge in Chickens and Mice, Veterinary Microbiology. (2022) 264, 10.1016/j.vetmic.2021.109306, 109306. 34923247

[55] Hu J. , Peng P. , and Li J. , et al.Single Dose of Bivalent H5 and H7 Influenza Virus-Like Particle Protects Chickens Against Highly Pathogenic H5N1 and H7N9 Avian Influenza Viruses, Frontiers in Veterinary Science. (2021) 8, 10.3389/fvets.2021.774630, 774630. 34859093 PMC8632145

[56] Lee D. H. , Park J. K. , and Lee Y. N. , et al.H9N2 Avian Influenza Virus-Like Particle Vaccine Provides Protective Immunity and a Strategy for the Differentiation of Infected From Vaccinated Animals, Vaccine. (2011) 29, no. 23, 4003–4007, 10.1016/j.vaccine.2011.03.067, 2-s2.0-79955660250. 21463681 PMC5555295

[57] Zhao Y. , Liu J. , and Peng C. , et al.Cross-Protection Against Homo and Heterologous Influenza Viruses via Intranasal Administration of an HA Chimeric Multiepitope Nanoparticle Vaccine, Journal of Nanobiotechnology. (2025) 23, no. 1, 10.1186/s12951-025-03122-6, 77. 39905416 PMC11792681

[58] Chen T. , Gao Y. , and Chen X. , et al.Self-Assembling Nanoparticle Vaccine Elicits a Robust Protective Immune Response Against Avian Influenza H5N6 Virus in Chickens, International Journal of Biological Macromolecules. (2025) 287, 10.1016/j.ijbiomac.2024.138405, 138405. 39643188

[59] Ren H. , Zhang B. , and Zhang X. , et al.Self-Assembling Nanoparticle Hemagglutinin Influenza Vaccines Induce High Antibody Response, International Journal of Molecular Sciences. (2024) 25, no. 13, 10.3390/ijms25137259, 7259. 39000366 PMC11241447

[60] Ding P. , Liu H. , and Zhu X. , et al.Thiolated Chitosan Encapsulation Constituted Mucoadhesive Nanovaccine Confers Broad Protection Against Divergent Influenza A Viruses, Carbohydrate Polymers. (2024) 328, 10.1016/j.carbpol.2023.121689, 121689. 38220319

[61] Singh S. M. , Alkie T. N. , Nagy E. , Kulkarni R. R. , Hodgins D. C. , and Sharif S. , Delivery of an Inactivated Avian Influenza Virus Vaccine Adjuvanted With Poly(D, L-lactic-co-glycolic acid) Encapsulated CpG ODN Induces Protective Immune Responses in Chickens, Vaccine. (2016) 34, no. 40, 4807–4813, 10.1016/j.vaccine.2016.08.009, 2-s2.0-84995527940. 27543454

[62] Singh S. M. , Alkie T. N. , and Abdelaziz K. T. , et al.Characterization of Immune Responses to an Inactivated Avian Influenza Virus Vaccine Adjuvanted With Nanoparticles Containing CpG ODN, Viral Immunology. (2016) 29, no. 5, 269–275, 10.1089/vim.2015.0144, 2-s2.0-84973441796. 27077969

[63] Kingstad-Bakke B. A. , Chandrasekar S. S. , and Phanse Y. , et al.Effective Mosaic-Based Nanovaccines Against Avian Influenza in Poultry, Vaccine. (2019) 37, no. 35, 5051–5058, 10.1016/j.vaccine.2019.06.077, 2-s2.0-85068452850. 31300285

[64] Ross K. A. , Loyd H. , and Wu W. , et al.Hemagglutinin-Based Polyanhydride Nanovaccines Against H5N1 Influenza Elicit Protective Virus Neutralizing Titers and Cell-Mediated Immunity, International journal of nanomedicine. (2015) 10, 229–243, 10.2147/IJN.S72264, 2-s2.0-84920140477. 25565816 PMC4284014

[65] Dehghan A. , Shahsavandi S. , and Jabalameli L. , Improvement Efficacy of Influenza Nanovaccine in Combination With Hemokinin-1 Molecular Adjuvant, Avicenna Journal of Medical Biotechnology. (2018) 10, no. 4, 208–213. 30555652 PMC6252024

[66] Zhang T. , Tian Y. , and Zhang X. , et al.Improved Cellular Immune Response Induced by Intranasal Boost Immunization With Chitosan Coated DNA Vaccine Against H9N2 Influenza Virus Challenge, Microbial Pathogenesis. (2024) 195, 10.1016/j.micpath.2024.106871, 106871. 39163919

[67] Furey C. , Scher G. , and Ye N. , et al.Development of a Nucleoside-Modified mRNA Vaccine Against Clade 2.3.4.4b H5 Highly Pathogenic Avian Influenza Virus, Nature Communications. (2024) 15, no. 1, 10.1038/s41467-024-48555-z, 4350. PMC11116520 38782954

[68] Cui X. , Vervaeke P. , and Gao Y. , et al.Immunogenicity and Biodistribution of Lipid Nanoparticle Formulated Self-Amplifying mRNA Vaccines Against H5 Avian Influenza, npj Vaccines. (2024) 9, no. 1, 10.1038/s41541-024-00932-x, 138. 39097672 PMC11298010

[69] Xu S. , Zhang B. , Yao J. , and Ruan W. , A New H9 Influenza Virus mRNA Vaccine Elicits Robust Protective Immunity Against Infection, Vaccine. (2023) 41, no. 18, 2905–2913, 10.1016/j.vaccine.2023.03.049. 37005103

[70] Abdelwahab W. M. , Auclair S. , and Borgogna T. , et al.Co-Delivery of a Novel Lipidated TLR7/8 Agonist and Hemagglutinin-Based Influenza Antigen Using Silica Nanoparticles Promotes Enhanced Immune Responses, Pharmaceutics. (2024) 16, no. 1, 10.3390/pharmaceutics16010107, 107. 38258117 PMC10819884

[71] Tao W. , Hurst B. L. , and Shakya A. K. , et al.Consensus M2e Peptide Conjugated to Gold Nanoparticles Confers Protection Against H1N1, H3N2 and H5N1 Influenza A Viruses, Antiviral Research. (2017) 141, 62–72, 10.1016/j.antiviral.2017.01.021, 2-s2.0-85013306254. 28161578 PMC5572660

[72] Motamedi-Sedeh F. , Saboorizadeh A. , Khalili I. , Sharbatdaran M. , Wijewardana V. , and Arbabi A. , Carboxymethyl Chitosan Bounded Iron Oxide Nanoparticles and Gamma-Irradiated Avian Influenza Subtype H9N2 Vaccine to Development of Immunity on Mouse and Chicken, Veterinary Medicine and Science. (2022) 8, no. 2, 626–634, 10.1002/vms3.680. 34878724 PMC8959295

[73] He J. , Liu Z. , and Jiang W. , et al.Immune-Adjuvant Activity of Lentinan-Modified Calcium Carbonate Microparticles on a H5N1 Vaccine, International Journal of Biological Macromolecules. (2020) 163, 1384–1392, 10.1016/j.ijbiomac.2020.08.005. 32758599

[74] Xu X. , Ding Z. , and Yuan Q. , et al.A Genotype VII Newcastle Disease Virus-Like Particles Confer Full Protection With Reduced Virus Load and Decreased Virus Shedding, Vaccine. (2019) 37, no. 3, 444–451, 10.1016/j.vaccine.2018.11.068, 2-s2.0-85058002984. 30545716

[75] Park J. K. , Lee D. H. , and Yuk S. S. , et al.Virus-Like Particle Vaccine Confers Protection Against a Lethal Newcastle Disease Virus Challenge in Chickens and Allows a Strategy of Differentiating Infected From Vaccinated Animals, Clinical and Vaccine Immunology. (2014) 21, no. 3, 360–365, 10.1128/CVI.00636-13, 2-s2.0-84896769763. 24403523 PMC3957659

[76] Shen H. , Xue C. , and Lv L. , et al.Assembly and Immunological Properties of a Bivalent Virus-Like Particle (VLP) for Avian Influenza and Newcastle Disease, Virus Research. (2013) 178, no. 2, 430–436, 10.1016/j.virusres.2013.09.009, 2-s2.0-84888206375. 24050994

[77] Ananda Kumar B. S. , Panickan S. , and Bindu S. , et al.Immunogenicity and Protective Efficacy of an Inactivated Newcastle Disease Virus Vaccine Encapsulated in Poly-(lactic-co-glycolic acid) Nanoparticles, Poultry Science. (2023) 102, no. 6, 10.1016/j.psj.2023.102679, 102679. PMC10160591 37116285

[78] Yang Y. , Xing R. , and Liu S. , et al.Chitosan, Hydroxypropyltrimethyl Ammonium Chloride Chitosan and Sulfated Chitosan Nanoparticles as Adjuvants for Inactivated Newcastle Disease Vaccine, Carbohydrate Polymers. (2020) 229, 10.1016/j.carbpol.2019.115423, 115423. 31826462

[79] Zhao K. , Sun B. , Shi C. , Sun Y. , Jin Z. , and Hu G. , Intranasal Immunization With O-2^′^-Hydroxypropyl Trimethyl Ammonium Chloride Chitosan Nanoparticles Loaded With Newcastle Disease Virus DNA Vaccine Enhances Mucosal Immune Response in Chickens, Journal of Nanobiotechnology. (2021) 19, no. 1, 10.1186/s12951-021-00983-5, 240. 34380522 PMC8359106

[80] Firouzamandi M. , Moeini H. , and Hosseini S. D. , et al.Preparation, Characterization, and in Ovo Vaccination of Dextran-Spermine Nanoparticle DNA Vaccine Coexpressing the Fusion and Hemagglutinin Genes Against Newcastle Disease, International Journal of Nanomedicine. (2016) 11, 259–267, 10.2147/IJN.S92225, 2-s2.0-84955084473. 26834470 PMC4716742

[81] Zhang J. , Ji Y. , Wang Z. , Jia Y. , and Zhu Q. , Effective Improvements to the Live-Attenuated Newcastle Disease Virus Vaccine by Polyethylenimine-Based Biomimetic Silicification, Vaccine. (2022) 40, no. 6, 886–896, 10.1016/j.vaccine.2021.12.054. 34991927

[82] Volkova M. A. , Irza A. V. , Chvala I. A. , Frolov S. F. , Drygin V. V. , and Kapczynski D. R. , Adjuvant Effects of Chitosan and Calcium Phosphate Particles in an Inactivated Newcastle Disease Vaccine, Avian Diseases. (2014) 58, no. 1, 46–52, 10.1637/10510-020413-Reg.1, 2-s2.0-84896361608. 24758112

[83] Sepotokele K. M. , O’Kennedy M. M. , Hayes M. C. , Wandrag D. B. R. , Smith P. , and Abolnik C. , Efficacy of a Plant-Produced Infectious Bronchitis Virus-Like Particle Vaccine in Specific Pathogen-Free Chickens, Poultry Science. (2023) 102, no. 10, 10.1016/j.psj.2023.102953, 102953. PMC10407904 37542940

[84] Li J. , Helal Z. H. , and Karch C. P. , et al.A Self-Adjuvanted Nanoparticle Based Vaccine Against Infectious Bronchitis Virus, PLoS ONE. (2018) 13, no. 9, 10.1371/journal.pone.0203771, 2-s2.0-85053297305, e0203771. 30216376 PMC6138407

[85] Lin S.-Y. , Yao B.-Y. , Hu C.-M. J. , and Chen H.-W. , Induction of Robust Immune Responses by CpG-ODN-Loaded Hollow Polymeric Nanoparticles for Antiviral and Vaccine Applications in Chickens, International Journal of Nanomedicine. (2020) 15, 3303–3318, 10.2147/IJN.S241492. 32494131 PMC7227821

[86] Lopes P. D. , Okino C. H. , and Fernando F. S. , et al.Inactivated Infectious Bronchitis Virus Vaccine Encapsulated in Chitosan Nanoparticles Induces Mucosal Immune Responses and Effective Protection Against Challenge, Vaccine. (2018) 36, no. 19, 2630–2636, 10.1016/j.vaccine.2018.03.065, 2-s2.0-85045124096. 29653848

[87] Bande F. , Arshad S. S. , and Bejo M. H. , et al.Development and Immunogenic Potentials of Chitosan-Saponin Encapsulated DNA Vaccine Against Avian Infectious Bronchitis Coronavirus, Microbial Pathogenesis. (2020) 149, 10.1016/j.micpath.2020.104560, 104560. 33068733 PMC7556284

[88] Chen H. W. , Huang C. Y. , and Lin S. Y. , et al.Synthetic Virus-Like Particles Prepared via Protein Corona Formation Enable Effective Vaccination in an Avian Model of Coronavirus Infection, Biomaterials. (2016) 106, 111–118, 10.1016/j.biomaterials.2016.08.018, 2-s2.0-84983071815. 27552321 PMC7112462

[89] Tufail S. , Shah M. A. , and Zafar M. , et al.Identification of Potent Epitopes on Hexon Capsid Protein and Their Evaluation as Vaccine Candidates Against Infections Caused by Members of Adenoviridae Family, Vaccine. (2021) 39, no. 27, 3560–3564, 10.1016/j.vaccine.2021.05.023. 34030897

[90] Tischer B. K. , Schumacher D. , and Beer M. , et al.A DNA Vaccine Containing an Infectious Marek’s Disease Virus Genome can Confer Protection Against Tumorigenic Marek’s Disease in Chickens, Journal of General Virology. (2002) 83, no. 10, 2367–2376, 10.1099/0022-1317-83-10-2367, 2-s2.0-0036787220. 12237417

[91] Fazel F. , Matsuyama-Kato A. , and Alizadeh M. , et al.A Marek’s Disease Virus Messenger RNA-Based Vaccine Modulates Local and Systemic Immune Responses in Chickens, Viruses. (2024) 16, no. 7, 10.3390/v16071156, 1156. 39066318 PMC11281610

[92] Li G. , Kuang H. , and Guo H. , et al.Development of a Recombinant VP2 Vaccine for the Prevention of Novel Variant Strains of Infectious Bursal Disease Virus, Avian Pathology. (2020) 49, no. 6, 557–571, 10.1080/03079457.2020.1791314. 32658552

[93] Wang Y. , Jiang N. , and Fan L. , et al.Development of a Viral-Like Particle Candidate Vaccine Against Novel Variant Infectious Bursal Disease Virus, Vaccines. (2021) 9, no. 2, 10.3390/vaccines9020142, 142. 33579020 PMC7916800

[94] Zhang C. , Liu J. , and Xing Z. , et al.PLGA Nanoparticle With *Amomum longiligulare* Polysaccharide 1 Increased the Immunogenicity of Infectious Bursal Disease Virus VP2 Protein, British Poultry Science. (2023) 64, no. 2, 176–184, 10.1080/00071668.2022.2154639. 36469700

[95] Taghizadeh M. S. , Niazi A. , and Afsharifar A. , Virus-Like Particles (VLPs): A Promising Platform for Combating Against Newcastle Disease Virus, Vaccine: X. (2024) 16, 10.1016/j.jvacx.2024.100440, 100440. 38283623 PMC10811427

[96] Yang Y.-H. , Tai C.-H. , Cheng D. , Wang Y.-F. , and Wang J.-R. , Investigation of Avian Influenza H5N6 Virus-Like Particles as a Broad-Spectrum Vaccine Candidate Against H5Nx Viruses, Viruses. (2022) 14, no. 5, 10.3390/v14050925, 925. 35632667 PMC9143382

[97] Kang H. J. , Chu K. B. , and Lee D. H. , et al.Influenza M2 Virus-Like Particle Vaccination Enhances Protection in Combination With Avian Influenza HA VLPs, PLoS ONE. (2019) 14, no. 6, 10.1371/journal.pone.0216871, 2-s2.0-85068984500, e0216871. 31246961 PMC6597044

[98] Kang S.-M. , Song J.-M. , Quan F.-S. , and Compans R. W. , Influenza Vaccines Based on Virus-Like Particles, Virus Research. (2009) 143, no. 2, 140–146, 10.1016/j.virusres.2009.04.005, 2-s2.0-68749113789. 19374929 PMC2753524

[99] Ninyio N. N. , Ho K. L. , Omar A. R. , Tan W. S. , Iqbal M. , and Mariatulqabtiah A. R. , Virus-Like Particle Vaccines: A Prospective Panacea Against an Avian Influenza Panzootic, Vaccines. (2020) 8, no. 4, 10.3390/vaccines8040694, 694. 33227887 PMC7712863

[100] Xu H. , Zhu S. , Govinden R. , and Chenia H. Y. , Multiple Vaccines and Strategies for Pandemic Preparedness of Avian Influenza Virus, Viruses. (2023) 15, no. 8, 10.3390/v15081694, 1694. 37632036 PMC10459121

[101] Kang H. J. , Chu K. B. , and Yoon K. W. , et al.Multiple Neuraminidase Containing Influenza Virus-Like Particle Vaccines Protect Mice From Avian and Human Influenza Virus Infection, Viruses. (2022) 14, no. 2, 10.3390/v14020429, 429. 35216022 PMC8875606

[102] Hu C. J. , Chien C. Y. , and Liu M. T. , et al.Multi-Antigen Avian Influenza a (H7N9) Virus-Like Particles: Particulate Characterizations and Immunogenicity Evaluation in Murine and Avian Models, BMC Biotechnology. (2017) 17, no. 1, 10.1186/s12896-016-0321-6, 2-s2.0-85008323013, 2. 28061848 PMC5219756

[103] McGinnes L. W. and Morrison T. G. , Newcastle Disease Virus-Like Particles: Preparation, Purification, Quantification, and Incorporation of Foreign Glycoproteins, Current Protocols in Microbiology. (2013) 30, no. 1, 1821–1822, 10.1002/9780471729259.mc1802s30. PMC4036627 24510891

[104] Qian J. , Xu X. , and Ding J. , et al.Newcastle Disease Virus-Like Particles Induce DC Maturation Through TLR4/NF-κB Pathway and Facilitate DC Migration by CCR7-CCL19/CCL21 Axis, Veterinary Microbiology. (2017) 203, 158–166, 10.1016/j.vetmic.2017.03.002, 2-s2.0-85015265391. 28619138

[105] McGinnes L. W. , Pantua H. , Laliberte J. P. , Gravel K. A. , Jain S. , and Morrison T. G. , Assembly and Biological and Immunological Properties of Newcastle Disease Virus-Like Particles, Journal of Virology. (2010) 84, no. 9, 4513–4523, 10.1128/JVI.01931-09, 2-s2.0-77950837713. 20181713 PMC2863756

[106] Xu X. , Qian J. , and Qin L. , et al.Chimeric Newcastle Disease Virus-Like Particles Containing DC-Binding Peptide-Fused Haemagglutinin Protect Chickens From Virulent Newcastle Disease Virus and H9N2 Avian Influenza Virus Challenge, Virologica Sinica. (2020) 35, no. 4, 455–467, 10.1007/s12250-020-00199-1. 32274680 PMC7462956

[107] Wu X. , Zhai X. , and Lai Y. , et al.Construction and Immunogenicity of Novel Chimeric Virus-Like Particles Bearing Antigens of Infectious Bronchitis Virus and Newcastle Disease Virus, Viruses. (2019) 11, no. 3, 10.3390/v11030254, 2-s2.0-85062953133, 254. 30871190 PMC6465995

[108] Smith T. , O’Kennedy M. M. , Ross C. S. , Lewis N. S. , and Abolnik C. , The Production of Newcastle Disease Virus-Like Particles in *Nicotiana benthamiana* as Potential Vaccines, Frontiers in Plant Science. (2023) 14, 10.3389/fpls.2023.1130910, 1130910. 36875611 PMC9978804

[109] Peyret H. , Steele J. F. C. , Jung J.-W. , Thuenemann E. C. , Meshcheriakova Y. , and Lomonossoff G. P. , Producing Vaccines Against Enveloped Viruses in Plants: Making the Impossible, Difficult, Vaccines. (2021) 9, no. 7, 10.3390/vaccines9070780, 780. 34358196 PMC8310165

[110] de Swart R. L. and Belov G. A. , Advantages and Challenges of Newcastle Disease Virus as a Vector for Respiratory Mucosal Vaccines, Current Opinion in Virology. (2023) 62, 10.1016/j.coviro.2023.101348, 101348. 37591130 PMC13262185

[111] Lv L. , Li X. , and Liu G. , et al.Production and Immunogenicity of Chimeric Virus-Like Particles Containing the Spike Glycoprotein of Infectious Bronchitis Virus, Journal of Veterinary Science. (2014) 15, no. 2, 209–216, 10.4142/jvs.2014.15.2.209, 2-s2.0-84902332626. 24378590 PMC4087222

[112] Sepotokele K. M. , O’Kennedy M. M. , Wandrag D. B. R. , and Abolnik C. , Optimization of Infectious Bronchitis Virus-Like Particle Expression in *Nicotiana benthamiana* as Potential Poultry Vaccines, PLoS ONE. (2023) 18, no. 7, 10.1371/journal.pone.0288970, e0288970. 37471377 PMC10358894

[113] Ji P. , Li T. , and Wu Y. , et al.Virus-Like Particle Vaccines of Infectious Bursal Disease Virus Expressed in *Escherichia coli* Are Highly Immunogenic and Protect Against Virulent Strain, Viruses. (2023) 15, no. 11, 10.3390/v15112178, 2178. 38005855 PMC10674347

[114] Lee H.-J. , Kim J.-Y. , Kye S.-J. , Seul H.-J. , Jung S.-C. , and Choi K.-S. , Efficient Self-Assembly and Protective Efficacy of Infectious Bursal Disease Virus-Like Particles by a Recombinant Baculovirus Co-Expressing Precursor Polyprotein and VP4, Virology Journal. (2015) 12, no. 1, 10.1186/s12985-015-0403-4, 2-s2.0-84945282194, 177. 26502988 PMC4621879

[115] Wu H. , Weng R. , and Li J. , et al.Self-Assembling Protein Nanoparticle Platform for Multivalent Antigen Delivery in Vaccine Development, International Journal of Pharmaceutics. (2025) 676, 10.1016/j.ijpharm.2025.125597, 125597. 40233885

[116] Xu S. , Sun C. , and Qian T. , et al.Animal Vaccine Revolution: Nanoparticle Adjuvants Open the Future of Vaccinology, Journal of Controlled Release. (2025) 383, 10.1016/j.jconrel.2025.113827, 113827. 40349784

[117] He L. , Pan R. , and Liang R. , et al.Nanomaterial Adjuvants for Veterinary Vaccines: Mechanisms and Applications, Research. (2025) 8, 10.34133/research.0761, 0761. 40636132 PMC12237591

[118] Kar U. , Khaleeq S. , and Garg P. , et al.Comparative Immunogenicity of Bacterially Expressed Soluble Trimers and Nanoparticle Displayed Influenza Hemagglutinin Stem Immunogens, Frontiers in Immunology. (2022) 13, 10.3389/fimmu.2022.890622, 890622. 35720346 PMC9204493

[119] Pascual E. , Mata C. P. , and Gomez-Blanco J. , et al.Structural Basis for the Development of Avian Virus Capsids that Display Influenza Virus Proteins and Induce Protective Immunity, Journal of Virology. (2015) 89, no. 5, 2563–2574, 10.1128/JVI.03025-14, 2-s2.0-84922998362. 25520499 PMC4325719

[120] Zhao K. , Li D. , and Shi C. , et al.Biodegradable Polymeric Nanoparticles as the Delivery Carrier for Drug, Current Drug Delivery. (2016) 13, no. 4, 494–499, 10.2174/156720181304160521004609, 2-s2.0-84973626348. 27230997

[121] Zaheer T. , Pal K. , and Zaheer I. , Topical Review on Nano-Vaccinology: Biochemical Promises and Key Challenges, Process Biochemistry. (2021) 100, 237–244, 10.1016/j.procbio.2020.09.028. 33013180 PMC7521878

[122] Gutjahr A. , Phelip C. , and Coolen A. L. , et al.Biodegradable Polymeric Nanoparticles-Based Vaccine Adjuvants for Lymph Nodes Targeting, Vaccines. (2016) 4, no. 4, 10.3390/vaccines4040034, 2-s2.0-85014350383, 34. 27754314 PMC5192354

[123] Alkie T. N. , Yitbarek A. , Taha-Abdelaziz K. , Astill J. , and Sharif S. , Characterization of Immunogenicity of Avian Influenza Antigens Encapsulated in PLGA Nanoparticles Following Mucosal and Subcutaneous Delivery in Chickens, PLoS ONE. (2018) 13, no. 11, 10.1371/journal.pone.0206324, 2-s2.0-85055903373, e0206324. 30383798 PMC6211703

[124] Zhao K. , Li W. , and Huang T. , et al.Preparation and Efficacy of Newcastle Disease Virus DNA Vaccine Encapsulated in PLGA Nanoparticles, PLoS ONE. (2013) 8, no. 12, 10.1371/journal.pone.0082648, 2-s2.0-84891308894, e82648. 24386106 PMC3873271

[125] Saravanakumar G. , Jo D.-G. , and Park J. H. , Polysaccharide-Based Nanoparticles: A Versatile Platform for Drug Delivery and Biomedical Imaging, Current Medicinal Chemistry. (2012) 19, no. 19, 3212–3229, 10.2174/092986712800784658, 2-s2.0-84863698864. 22612705

[126] Jiang Y. , Qi S. , and Mao C. , Polysaccharide Nanoparticles as Potential Immune Adjuvants: Mechanism and Function, Acta Pharmaceutica Sinica B. (2025) 15, no. 4, 1796–1815, 10.1016/j.apsb.2025.03.006. 40486863 PMC12137980

[127] Wusiman A. , He J. , and Zhu T. , et al.Macrophage Immunomodulatory Activity of the Cationic Polymer Modified PLGA Nanoparticles Encapsulating Alhagi Honey Polysaccharide, International Journal of Biological Macromolecules. (2019) 134, 730–739, 10.1016/j.ijbiomac.2019.05.038, 2-s2.0-85065883662. 31071396

[128] Zhao K. , Zhang Y. , and Zhang X. , et al.Chitosan-Coated Poly(lactic-co-glycolic) Acid Nanoparticles as an Efficient Delivery System for Newcastle Disease Virus DNA Vaccine, International Journal of Nanomedicine. (2014) 9, 4609–4619, 10.2147/IJN.S70633, 2-s2.0-84907881420. 25356070 PMC4207079

[129] Bamberger D. , Hobernik D. , Konhäuser M. , Bros M. , and Wich P. R. , Surface Modification of Polysaccharide-Based Nanoparticles With PEG and Dextran and the Effects on Immune Cell Binding and Stimulatory Characteristics, Molecular Pharmaceutics. (2017) 14, no. 12, 4403–4416, 10.1021/acs.molpharmaceut.7b00507, 2-s2.0-85037660380. 29063757

[130] Li X. , Xing R. , and Xu C. , et al.Immunostimulatory Effect of Chitosan and Quaternary Chitosan: A Review of Potential Vaccine Adjuvants, Carbohydrate Polymers. (2021) 264, 10.1016/j.carbpol.2021.118050, 118050. 33910752

[131] Li D. , Fu D. , and Kang H. , et al.Advances and Potential Applications of Chitosan Nanoparticles as a Delivery Carrier for the Mucosal Immunity of Vaccine, Current Drug Delivery. (2017) 14, no. 1, 27–35, 10.2174/1567201813666160804121123, 2-s2.0-85010281643. 27494157

[132] Bolhassani A. , Lipid-Based Delivery Systems in Development of Genetic and Subunit Vaccines, Molecular Biotechnology. (2023) 65, no. 5, 669–698, 10.1007/s12033-022-00624-8. 36462102 PMC9734811

[133] Pardi N. , Hogan M. J. , Porter F. W. , and Weissman D. , mRNA Vaccines—A New Era in Vaccinology, Nature Reviews Drug Discovery. (2018) 17, no. 4, 261–279, 10.1038/nrd.2017.243, 2-s2.0-85044634269. 29326426 PMC5906799

[134] Hou X. , Zaks T. , Langer R. , and Dong Y. , Lipid Nanoparticles for mRNA Delivery, Nature Reviews Materials. (2021) 6, no. 12, 1078–1094, 10.1038/s41578-021-00358-0. 34394960 PMC8353930

[135] Tenchov R. , Bird R. , Curtze A. E. , and Zhou Q. , Lipid Nanoparticles Horizontal Line From Liposomes to mRNA Vaccine Delivery, a Landscape of Research Diversity and Advancement, ACS Nano. (2021) 15, no. 11, 16982–17015, 10.1021/acsnano.1c04996. 34181394

[136] Le T. , Sun C. , Chang J. , Zhang G. , and Yin X. , mRNA Vaccine Development for Emerging Animal and Zoonotic Diseases, Viruses. (2022) 14, no. 2, 10.3390/v14020401. PMC8877136 35215994

[137] Reichmuth A. M. , Oberli M. A. , Jaklenec A. , Langer R. , and Blankschtein D. , mRNA Vaccine Delivery Using Lipid Nanoparticles, Therapeutic Delivery. (2016) 7, no. 5, 319–334, 10.4155/tde-2016-0006, 2-s2.0-84964930086. 27075952 PMC5439223

[138] Kawai A. , Shimizu T. , and Tanaka H. , et al.Low-Inflammatory Lipid Nanoparticle-Based mRNA Vaccine Elicits Protective Immunity Against H5N1 Influenza Virus With Reduced Adverse Reactions, Molecular Therapy. (2025) 33, no. 2, 529–547, 10.1016/j.ymthe.2024.12.032. 39690742 PMC11852987

[139] Cheng J. , Wen S. , and Wang S. , et al.gp85 Protein Vaccine Adjuvanted With Silica Nanoparticles Against ALV-J in Chickens, Vaccine. (2017) 35, no. 2, 293–298, 10.1016/j.vaccine.2016.11.077, 2-s2.0-85006973386. 27912987

[140] Wang C. , Zhu W. , Luo Y. , and Wang B.-Z. , Gold Nanoparticles Conjugating Recombinant Influenza Hemagglutinin Trimers and Flagellin Enhanced Mucosal Cellular Immunity, Nanomedicine. (2018) 14, no. 4, 1349–1360, 10.1016/j.nano.2018.03.007, 2-s2.0-85046649452. 29649593 PMC6177327

[141] Salazar-González J. A. , González-Ortega O. , and Rosales-Mendoza S. , Gold Nanoparticles and Vaccine Development, Expert Review of Vaccines. (2015) 14, no. 9, 1197–1211, 10.1586/14760584.2015.1064772, 2-s2.0-84939467744. 26152550

[142] Sengupta A. , Azharuddin M. , Al-Otaibi N. , and Hinkula J. , Efficacy and Immune Response Elicited by Gold Nanoparticle- Based Nanovaccines Against Infectious Diseases, Vaccines. (2022) 10, no. 4, 10.3390/vaccines10040505, 505. 35455254 PMC9030786

[143] Viswanathan K. , Gopinath V. P. , and Raj G. D. , Formulation of Newcastle Disease Virus Coupled Calcium Phosphate Nanoparticles: An Effective Strategy for Oculonasal Delivery to Chicken, Colloids and Surfaces B: Biointerfaces. (2014) 116, 9–16, 10.1016/j.colsurfb.2013.12.017, 2-s2.0-84892511634. 24441177

[144] Sadr S. , Lotfalizadeh N. , Ghafouri S. A. , Delrobaei M. , Komeili N. , and Hajjafari A. , Nanotechnology Innovations for Increasing the Productivity of Poultry and the Prospective of Nanobiosensors, Veterinary Medicine and Science. (2023) 9, no. 5, 2118–2131, 10.1002/vms3.1193. 37433046 PMC10508580

[145] Tan H. , Mao K. , and Cong X. , et al.In Vivo Immune Adjuvant Effects of CaCO_3_ Nanoparticles Through Intracellular Ca^2+^ Concentration Regulation, ACS Appl Mater Interfaces. (2023) 15, no. 33, 39157–39166. 37553750 10.1021/acsami.3c07306

[146] Shen C. C. , Liang H. J. , Wang C. C. , Liao M. H. , and Jan T. R. , Iron Oxide Nanoparticles Suppressed T helper 1 Cell-Mediated Immunity in a Murine Model of Delayed-Type Hypersensitivity, International Journal of Nanomedicine. (2012) 7, 2729–2737. 22701318 10.2147/IJN.S31054 PMC3373298

[147] Ahmad A. , Ansari M. M. , and Kumar A. , et al.Comparative Acute Intravenous Toxicity Study of Triple Polymer-Layered Magnetic Nanoparticles With Bare Magnetic Nanoparticles in Swiss Albino Mice, Nanotoxicology. (2020) 14, no. 10, 1362–1380, 10.1080/17435390.2020.1829144. 33040614

[148] Neto L. M. M. , Zufelato N. , and de Sousa-Junior A. A. , et al.Specific T Cell Induction Using Iron Oxide Based Nanoparticles as Subunit Vaccine Adjuvant, Human Vaccines & Immunotherapeutics. (2018) 14, no. 11, 2786–2801, 10.1080/21645515.2018.1489192, 2-s2.0-85049799829. 29913109 PMC6314432

[149] Chaves L. L. , Dourado D. , and Prunache I. B. , et al.Nanocarriers of Antigen Proteins for Vaccine Delivery, International Journal of Pharmaceutics. (2024) 659, 10.1016/j.ijpharm.2024.124162, 124162. 38663646

[150] Li X. , Wang X. , and Ito A. , Tailoring Inorganic Nanoadjuvants towards Next-Generation Vaccines, Chemical Society Reviews. (2018) 47, no. 13, 4954–4980, 10.1039/C8CS00028J, 2-s2.0-85049412230. 29911725

[151] Underwood C. and van Eps A. W. , Nanomedicine and Veterinary Science: The Reality and the Practicality, The Veterinary Journal. (2012) 193, no. 1, 12–23, 10.1016/j.tvjl.2012.01.002, 2-s2.0-84863778187. 22365842

[152] Kheirollahpour M. , Mehrabi M. , Dounighi N. M. , Mohammadi M. , and Masoudi A. , Nanoparticles and Vaccine Development, Pharmaceutical Nanotechnology. (2020) 8, no. 1, 6–21, 10.2174/2211738507666191024162042. 31647394

[153] Nguyen B. and Tolia N. H. , Protein-Based Antigen Presentation Platforms for Nanoparticle Vaccines, npj Vaccines. (2021) 6, no. 1, 10.1038/s41541-021-00330-7, 70. 33986287 PMC8119681

[154] Anderluzzi G. , Lou G. , and Woods S. , et al.The Role of Nanoparticle Format and Route of Administration on Self-Amplifying mRNA Vaccine Potency, Journal of Controlled Release. (2022) 342, 388–399, 10.1016/j.jconrel.2021.12.008. 34896446 PMC8660137

[155] Mir M. , Ahmed N. , and Rehman A. U. , Recent Applications of PLGA Based Nanostructures in Drug Delivery, Colloids and Surfaces B: Biointerfaces. (2017) 159, 217–231, 10.1016/j.colsurfb.2017.07.038, 2-s2.0-85026826728. 28797972

[156] Liu Y. , Crawford B. M. , and Vo-Dinh T. , Gold Nanoparticles-Mediated Photothermal Therapy and Immunotherapy, Immunotherapy. (2018) 10, no. 13, 1175–1188, 10.2217/imt-2018-0029, 2-s2.0-85053868919. 30236026

[157] Yusibov V. , Streatfield S. J. , and Kushnir N. , Clinical Development of Plant-Produced Recombinant Pharmaceuticals: Vaccines, Antibodies and Beyond, Human Vaccines. (2011) 7, no. 3, 313–321, 10.4161/hv.7.3.14207, 2-s2.0-79953697416. 21346417

[158] Peternel S. and Komel R. , Isolation of Biologically Active Nanomaterial (Inclusion Bodies) From Bacterial Cells, Microbial Cell Factories. (2010) 9, no. 1, 10.1186/1475-2859-9-66, 2-s2.0-77956472225, 66. 20831775 PMC2944166

[159] Joshi S. , Jindal P. , and Gautam S. , et al.Mini Review on the Lyophilization: A Basic Requirement for Formulation Development and Stability Modifier, ASSAY and Drug Development Technologies. (2025) 23, no. 4, 180–194, 10.1089/adt.2024.122. 40008995

[160] Souto E. B. , Blanco-Llamero C. , and Krambeck K. , et al.Regulatory Insights into Nanomedicine and Gene Vaccine Innovation: Safety Assessment, Challenges, and Regulatory Perspectives, Acta Biomaterialia. (2024) 180, 1–17, 10.1016/j.actbio.2024.04.010. 38604468

[161] Atlas R. M. and Maloy S. , Atlas R. M. and Maloy S. , The Future of One Health, One Health, 2014, John Wiley & Sons, Ltd, 303–306, 10.1128/9781555818432.ch20.

[162] Kirk R. G. W. , Recovering *The Principles of Humane Experimental Technique*: The 3Rs and the Human Essence of Animal Research, Science, Technology, & Human Values. (2018) 43, no. 4, 622–648, 10.1177/0162243917726579, 2-s2.0-85048948325. PMC6027778 30008492

[163] Strech D. and Dirnagl U. , 3Rs Missing: Animal Research Without Scientific Value Is Unethical, BMJ Open Science. (2019) 3, no. 1, 10.1136/bmjos-2018-000048. PMC8647585 35047678

[164] Adamson S. , Marich A. , and Roth I. , One Health in NSW: Coordination of Human and Animal Health Sector Management of Zoonoses of Public Health Significance, NSW Public Health Bulletin. (2011) 22, no. 6, 105–112. 21781617 10.1071/NB11003

